# Knockout mice with pituitary malformations help identify human cases of hypopituitarism

**DOI:** 10.1186/s13073-024-01347-y

**Published:** 2024-05-31

**Authors:** Julian Martinez-Mayer, Michelle L. Brinkmeier, Sean P. O’Connell, Arnold Ukagwu, Marcelo A. Marti, Mirta Miras, Maria V. Forclaz, Maria G. Benzrihen, Leonard Y. M. Cheung, Sally A. Camper, Buffy S. Ellsworth, Lori T. Raetzman, Maria I. Pérez-Millán, Shannon W. Davis

**Affiliations:** 1 Institute of Biosciences, Biotechnology and Translational Biology (iB3), University of Buenos Aires, Intendente Güiraldes 2160, Ciudad Universitaria, C1428EGA Buenos Aires, Argentina; 2 Department of Human Genetics, University of Michigan, 1241 Catherine St., Ann Arbor, MI 48109-5618 USA; 3 Department of Biological Sciences, University of South Carolina, 715 Sumter St., Columbia, SC 29208 USA; 4 Department of Physiology, Southern Illinois University, 1135 Lincoln Dr, Carbondale, IL 62901 USA; 5 Instituto de Química Biológica de La Facultad de Ciencias Exactas y Naturales (IQUIBICEN), Universidad de Buenos Aires, Buenos Aires, Argentina; 6 Hospital De Niños de La Santísima Trinidad, Córdoba, Argentina; 7 Servicio de Endocrinología, Hospital Posadas, Buenos Aires, Argentina; 8 Department of Physiology and Biophyscis, Renaissance School of Medicine, Stony Brook University, Stony Brook, NY 11794 USA; 9 Department of Molecular and Integrative Physiology, University of Illinois, Champaign-Urbana, Urbana, IL 61801 USA

**Keywords:** Pituitary, Hypothalamus, Growth hormone, Cleft palate, Holoprosencephaly, Septo-optic dysplasia, International Mouse Phenotyping Consortium (IMPC), *MORC2*, *SETD5*, Development

## Abstract

**Background:**

Congenital hypopituitarism (CH) and its associated syndromes, septo-optic dysplasia (SOD) and holoprosencephaly (HPE), are midline defects that cause significant morbidity for affected people. Variants in 67 genes are associated with CH, but a vast majority of CH cases lack a genetic diagnosis. Whole exome and whole genome sequencing of CH patients identifies sequence variants in genes known to cause CH, and in new candidate genes, but many of these are variants of uncertain significance (VUS).

**Methods:**

The International Mouse Phenotyping Consortium (IMPC) is an effort to establish gene function by knocking-out all genes in the mouse genome and generating corresponding phenotype data. We used mouse embryonic imaging data generated by the Deciphering Mechanisms of Developmental Disorders (DMDD) project to screen 209 embryonic lethal and sub-viable knockout mouse lines for pituitary malformations.

**Results:**

Of the 209 knockout mouse lines, we identified 51 that have embryonic pituitary malformations. These genes not only represent new candidates for CH, but also reveal new molecular pathways not previously associated with pituitary organogenesis. We used this list of candidate genes to mine whole exome sequencing data of a cohort of patients with CH, and we identified variants in two unrelated cases for two genes, *MORC2* and *SETD5*, with CH and other syndromic features.

**Conclusions:**

The screening and analysis of IMPC phenotyping data provide proof-of-principle that recessive lethal mouse mutants generated by the knockout mouse project are an excellent source of candidate genes for congenital hypopituitarism in children.

**Supplementary Information:**

The online version contains supplementary material available at 10.1186/s13073-024-01347-y.

## Background

The hypothalamus and pituitary gland are essential for regulating growth, homeostasis, fertility, water balance, and the stress response. These physiological processes are regulated by releasing hormones from the hypothalamus and hormones from the posterior and anterior lobes of the pituitary gland. People with CH have increased morbidity and mortality resulting from a loss of one or more pituitary hormones. The most common form of CH is combined pituitary hormone deficiency (CPHD), which is characterized by loss of growth hormone and at least one other pituitary hormone. CH can occur in isolation or as part of a syndrome with other midline defects, including HPE and SOD. HPE patients have variable defects in the forebrain, eyes, and pituitary, and severe cases are embryonic lethal. The triad of features diagnostic of SOD include optic nerve hypoplasia, midline brain abnormalities, and CH. Patients diagnosed with CH, but not HPE or SOD, sometimes have features associated with those disorders, including vision, hearing, and/or brain anomalies. In a recent study, 65% of the CPHD and isolated growth hormone deficiency (IGHD) patients had other syndromic features [1]. The genetic causes of these birth defects are highly overlapping. As such, they can be considered as a spectrum of related disorders and constitute an important group of structural birth defects that cause significant morbidity and life-long consequences for quality of life and care.

The first genes discovered to cause CPHD were pituitary transcription factors that were previously identified in mice, act early in pituitary organogenesis, and affect multiple hormone-producing cell types. These include *POU1F1*, *HESX1*, *PROP1*, *LHX3*, and *LHX4*, which were discovered in 1992–2001 [2]. Candidate gene screening for these five genes accounts for approximately 10–12% of cases worldwide, although the frequency varies by ethnicity [3–5]. By 2012, twenty-two different genes were implicated in CPHD. As whole exome sequencing was implemented, many more genes were identified, reaching 30 by 2014 and 67 by 2023 [6]. Table 1 contains 70 genes associated with CH, grouped according to major functional categories: epigenetic and transcriptional regulators, RNA regulation and translation, protein and metabolic pathways, and cell signaling or membrane and extracellular matrix. For some of these genes, there is insufficient evidence to conclude that the variants are pathogenic or likely pathogenic according to the American College of Medical Genetics guidelines [7]. Functional studies in mice or cell culture, or identification of additional unrelated families with likely pathogenic variants in the same gene could strengthen the implication of disease causality. The Online Mendelian Inheritance in Man (OMIM) link for each gene is provided along with additional human disorders associated with each gene. Mutations in most of these genes can cause complex phenotypes, as only 13% of the genes (9/70) are only associated with growth hormone deficiency, CPHD, or hypogonadotropic hypogonadism (HH) to date.

**Table 1:** Human genes implicated in hypothalamic-pituitary abnormalities

Gene	Description	OMIM ID	Human disorder
**Epigenetic and transcriptional regulators**			
*ARID1B*	AT-rich interaction domain-containing protein 1b, DNA-dependent ATPase	614,556	Coffin-Siris syndrome, intellectual disability, nail, hair, craniofacial abnormalities
*ARNT2*	Aryl hydrocarbon receptor nuclear translocator	606,036	CPHD, frontotemporal hypoplasia, developmental delay, blindness, kidney abnormalities
*CHD7*	Chromodomain helicase DNA binding protein 7	608,892	HH, Charge syndrome
*FOXA2*	Forkhead Box A2 (HNF3B)	600,288	GHD, CPHD, craniofacial abnormalities
*FOXL2*	Forkhead transcription factor	605,597	Blepharophimosis, epicanthus inversus, ptosis, premature ovarian failure
*GLI2*	GLI_Kruppel family member 2 responsive to SHH	165,230	Culler-Jones syndrome, HPE
*GLI3*	GLI_Kruppel family member 3 responsive to SHH	165,240	Greig cephalopolysyndactyly, Pallister Hall, pre and post-axial polydactyly
*GLI4*	GLI-Kruppel family member 4	165,280	Nothing in OMIM about disease per se
*HESX1*	homeobox gene expressed in ES cells	601,802	GHD, CPHD, SOD
*HMGA2*	High mobility group AT-Hook2	600,698	GHD, growth plate, Russell Silver Syndrome
*HNF1A*	HNF1 homeobox A	142,410	Diabetes, adenoma, carcinoma
*LHX3*	LIM homeobox gene 3	600,577	CPHD, hearing loss, rigid cervical spine
*LHX4*	LIM homeobox gene 4	602,146	CPHD, cerebellar defects
*NFKB2*	Nuclear factor Kappa-B subunit 2, cytokine responsive nuclear translocation	164,012	Common, variable, immunodeficiency
*NKX2.1*	NK2 homeobox 1	600,635	Chorea, choreoathetosis, hypothyroidism, neonatal respiratory distress
*OTX2*	Orthodenticle homolog 2	600,037	CPHD, microphthalmia, retinal dystrophy
*PAX6*	Paired homeodomain transcription factor 6	607,108	Aniridia, anterior segment dysgenesis, cataract, optic nerve hypoplasia
*POU1F1*	POU domain class 1 transcription factor	173,110	GHD, CPHD
*PROP1*	Paired homeodomain transcription factor	601,538	CPHD
*RAX*	Retina and anterior neural fold homeobox gene	601,881	Syndromic microphthalmia
*SIX1*	Sine oculis homeobox homolog 1	601,205	Branchio-otic syndrome, deafness
*SIX5*	Sine oculis homeobox homolog 5	600,963	Branchio-oto-renal syndrome
*SIX6*	Sine oculis homeobox homolog 6	606,326	Optic disc anomalies with retinal and/or macular dystrophy
*SMCHD1*	Structural maintenance of chromosomes flexible hinge domain protein 1	614,982	Bosma arhinia microphthalmia syndrome, fascioscapulohumeral muscular dystrophy
*SOX2*	SRY-related HMG-Box 2	184,429	Syndromic microphthalmia, optic nerve hypoplasia, CNS abnormalities
*SOX3*	SRY-related HMG-Box 3	313,430	IGHD, CPHD, Intellectual disability
*TCF7L1*	TCF/LEF family, high mobility group DNA binding domain	604,652	Optic nerve hypoplasia, corpus callosum, absent posterior pituitary
*TGIF1*	Transforming growth factor beta-induced factor, repressor of retinoid, TGF beta signaling	602,630	HPE
*ZIC2*	ZIC family member 2, interacts with TCF7L2 and inhibits WNT signaling	603,073	HPE
*ZSWIM6*	Zinc finger SWIM-type containing 6	615,951	Acromelic frontonasal dysostosis, neurodevelopmental disorder
**RNA regulation, translation**			
*EIF2S3*	Eukaryotic translation initiation factor 2, subunit 3	300,161	Mehmo syndrome: intellectual disability, hypopituitarism, midline defects
*HNRNPU*	Heterogeneous nuclear ribonucleoprotein U	602,869	Developmental and epileptic encephalopathy
*MIR17HG*	MicroRNA cluster miR-17-92a-1 host gene	609,415	Feingold syndrome, empty sella
*NONO*	Non-POU domain containing octamer binding protein, human splicing family	300,084	Intellectual disability
*POLR3A*	RNA polymerase 3 DNA-directed peptide A	614,258	HH, hypomyelinating leukodystrophy, oligodontia, Wiedemann_Rautenstrauch syndrome
*RBM28*	RNA binding motif protein 28, spliceosome component	612,074	Alopecia, neurologic defects, endocrinopathy syndrome
**Protein processing, trafficking, degradation, metabolic**			
*B3GAT3*	Beta-1,3-glucuronyltransferase 3	606,374	Larsen-like syndrome (short stature, skeletal deformities, and congenital heart defects)
*BRAF*	B-raf proto-oncogene, serine-threonine kinase	164,757	Noonan, LEOPARD, Cardiofaciocutaneous syndromes, cancers
*HID1*	Downregulated in multiple cancers, trans-Golgi network vesicle trafficking	605,752	Developmental epileptic encephalopathy 105 with hypopituitarism
*MAGEL2*	MAGE family member L2, enhances ubiquitin ligase activity	605,283	Schaaf-Yang syndrome
*PCSK1*	Proprotein convertase, subtilisin/kexin type 1 (PC1)	162,150	Endocrinopathy
*PNPLA6*	Patatin-like phospholipase domain-containing protein. 6	603,197	Laurence-Moon, Bocher-Neuhauser, Oliver-McFarlane syndromes, spastic paraplegia 39
*RNPC3*	RNA-binding region (RNP1, RRM)- containing 3, U11/U12-65 K protein, spliceosome	618,016	
*SLC15A4*	Solute carrier family 15, oligopeptide transporter	615,806	CPHD
*SLC20A1*	Solute carrier family 20, phosphate transporter	137,570	CPHD
*SPINK5*	Serine protease inhibitor, kazal type 5	256,500	Netherton syndrome
*SPR*	Sepiapterin reductase	182,125	Dopa-responsive dystonia
**Cell signaling, cilia, transmembrane, extracellular matrix**			
*BMP2*	Bone morphogenetic protein 2A	112,261	Brachydactyly, short stature, facial dysmorphism, skeletal, cardiac abnormalities
*BMP4*	Bone morphogenetic protein 4	112,262	Syndromic microphthalmia, orofacial cleft
*CDON*	Cell adhesion molecule downregulated by oncogenes, binds SHH	608,707	HPE
*FGF8*	Fibroblast growth factor 8	600,483	HH
*FGFR1*	Fibroblast growth factor receptor 1	136,350	HH, Hartsfield, Jackson-Weiss, Pfeiffer syndromes, encephalocraniocutaneous lipomatosis
*GPR161*	G protein-coupled receptor 161	612,250	Medulloblastoma predisposition syndrome
*HHIP*	Hedgehog interacting protein	606,178	HPE, CPHD, short stature QTL
*IFT172*	Homolog of intraflagellar transport 172	607,386	Bardet-Biedl syndrome, retinitis pigmentosa, short-rib thoracic dysplasia, polydactyly
*IGSF1*	Ig superfamily member 1	300,137	Central hypothyroidism and testicular enlargement
*IGSF10*	Ig superfamily member 10, neuronal migration	617,351	Delayed puberty without HH
*ANOS1*	Kallmann syndrome gene (ANOS1), cell adhesion molecule, neuronal migration	300,836	HH
*KCNQ1*	Voltage-gated potassium channel subfamily 1	607,542	Jervell and Lange-Nielsen syndrome, atrial fibrillation, long and short QT syndrome
*L1CAM*	L1 cell adhesion molecule, Ig superfamily	308,840	MASA, CRASH syndromes, hydrocephalus, intestinal obstruction, corpus callosum defect
*LAMB2*	Laminin beta 2	150,325	Pierson and Nephrotic syndrome, eye abnormalities
*PROKR2*	Prokineticin receptor 2, G protein coupled receptor	607,122	HH
*ROBO1*	Roundabout guidance receptor 1, neural cell adhesion molecule in Ig superfamily	602,430	CPHD, PSIS, craniofacial and ocular abnormalities
*SEMA3A*	Semaphorin 3A, neuronal migration, and circuitry	603,961	HH
*SHH*	Sonic hedgehog signaling molecule	600,725	HPE, microphthalmia with coloboma, schizencephaly, central incisor
*TBC1D32*	TBC1 domain family member 32, SHH signaling, stabilizes cell cycle-related kinase	615,867	VUS — orofacial digital syndrome with absent pituitary gland
*TMEM67*	Transmembrane protein 67, affects cilium formation (MKS3)	609,884	Meckel, Joubert, COACH, and RHYNS syndrome, nephronophthisis
*WDR11*	WD repeat domain 11, defective ciliogenesis and hedgehog signaling	606,417	HH
**Unknown/other**			
*NBPF9*	Neuroblastoma breakpoint family member 9, segmental duplications on HSA 21	613,999	Developmental and neurogenetic diseases
*BLM*	BLM recQ-like helicase, genome stability	210,900	Bloom syndrome, GHD

*Abbreviations*: *GHD* growth hormone deficiency, *CPHD* combined pituitary hormone deficiency, *HPE* holoprosencephaly, *SOD* septo-optic dysplasia, *HH* hypogonadotropic hypogonadism, *PSIS* pituitary stalk interruption syndrome

The rate of CH discovery shows no signs of leveling off, suggesting that more genes will be implicated in this disorder. Understanding the genetic causes of these disorders is challenging for many reasons. In addition to the high degree of genetic heterogeneity and variable clinical presentation, 57% of the genetic variants are dominant with incomplete penetrance and most cases are sporadic (Table 1) [2, 8–10]. This makes it difficult to be certain about causality when novel genes or variants are discovered. Because most mouse models of CPHD have been excellent predictors of the human phenotype, genetically engineered mice offer an important opportunity to improve our understanding of the genetic basis for CPHD and related disorders.

**Fig. 1:**
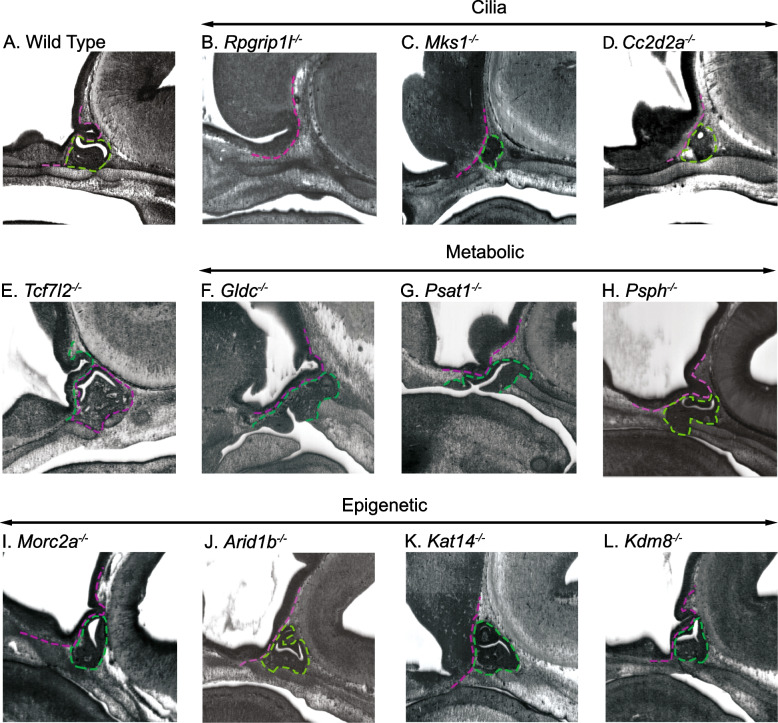
Pituitary gland malformations observed in candidate CH genes. Sagittal images captured from the DMDD HREM stackviewer for a e14.5 **A** wild type embryo and homozygous null embryos for **B ***Rpgrip1l*, **C***Mks1*, **D ***Cc2d2a*, **E ***Tcf7l2*, **F ***Gldc*, **G ***Psat1*, **H ***Psph*, **I ***Morc2a*, **J ***Arid1b*, **K ***Kat14*, and **L ***Kdm8*. In all images, the pituitary anterior and intermediate lobes are outlined in green and the basal side of the ventral diencephalon near the pituitary anterior lobe is outlined in magenta

In addition to being an excellent gene discovery model for CPHD, the mouse model system has been instrumental in formulating our general knowledge of mammalian pituitary gland organogenesis. The anterior lobe of the pituitary gland is generated from a cranial placode that forms in the oral ectoderm. This adenohypophyseal placode is induced by paracrine signals emanating from an organizing center in the ventral diencephalon [11, 12]. The adenohypophyseal placode will form an epithelial structure, known as Rathke’s pouch at e10.5, which contains the SOX2 expressing stem cells for both the intermediate lobe and anterior lobe [13]. The intermediate lobe and anterior lobe are in close juxtaposition throughout development [14], but they appear to be separated by a cleft in paraffin sections due to tissue shrinkage during processing. In histological sections, this apparent scissure has a characteristic sigmoidal shape at e14.5 (Fig. 1A). The dorsal side of the cleft, adjacent to the posterior lobe, will form the mouse intermediate lobe, a structure that does not remain distinct in humans [15]. The mouse intermediate lobe is enriched in melanotropes that process proopiomelanocortin (POMC) into melanocyte-stimulating hormone (MSH). The ventral side of the cleft is adjacent to the anterior lobe, where specialized cells reside that produce growth hormone (GH), thyroid stimulating hormone (TSH), prolactin (PRL), luteinizing hormone (LH), follicle-stimulating hormone (FSH), and adrenocorticotropic hormone (ACTH). During embryogenesis, the epithelial cells around the cleft are proliferative but then exit the cell cycle and perform an epithelial-to-mesenchymal-like transition to generate the parenchyma of the anterior lobe. Some of the cells that remain adjacent to the cleft retain their multipotency as pituitary stem cells [16].

IMPC is an international effort to phenotype mice generated by the International Mouse Knockout Consortium (IMKC). The IMPC is comprised of many phenotyping centers that have developed high throughput pipelines for characterizing, annotating, and disseminating the phenotypes associated with the null alleles for the targeted genes. One such center, Deciphering the Mechanisms of Developmental Disorders (DMDD), characterized the embryonic lethal and sub-viable phenotypes associated with 209 targeted genes generated in the UK [17, 18]. The DMDD pipeline involved sectioning embryonic lethal embryos and their wild-type littermates, mostly at the embryonic day of development 14.5 (e14.5), using high-resolution episcopic microscopy (HREM) to take high-resolution images of each section [17, 18]. The images were then used to generate a 3-dimensional model of each embryo that can be interrogated at all internal locations in three planes of section.

Using the data available from DMDD, we sought to identify novel genes that cause pituitary malformations. Using both the phenotypic annotations and available imagery, we screened the 209 genes for pituitary defects, identifying 51 genes, only one of which was known to function in pituitary gland organogenesis in mice [19]. This suggests that pituitary-hypothalamic malformations are a common component of mouse, recessive embryonic lethal strains. Furthermore, we present two human cases of CH-carrying variants in two of these genes, further cementing their status as candidate genes for congenital hypopituitarism.

## Methods

### Database searches

The DMDD website was accessed to obtain HREM data for all available 209 knockout lines generated by the Wellcome Trust Sanger Institute Mouse Genetics Project [17, 18]. We analyzed sagittal sections for mutants from all available knockout lines and identified 51 genes with pituitary malformations. Of those 51 lines only 15 had been annotated with pituitary malformations according to the DMDD table of annotated malformations (searched for the key words pituitary, adenohypophysis, and neurohypophysis). Screenshots of the sagittal sections were taken to document the malformations.

Each candidate gene was searched in the Online Mendelian Inheritance in Man (OMIM) database to identify associations with human disease. The candidate genes were also queried at GenePaint for RNA in situ hybridization data [20–22]. Images for midsagittal sections were downloaded, cropped, and levels adjusted in Photoshop to visualize expression in and around the pituitary gland.

We used the Database for Annotation, Visualization, and Integrated Discovery (DAVID) to identify the Gene Ontology (GO) terms associated with the list of known CH genes from Table 1 and each candidate gene from Table 2 [23, 24]. The full list of GO terms is presented in Additional File 1: Table S1. The gene lists were submitted as lists of official gene symbols for *Homo sapiens,* and the “Functional Annotation Tool” was selected using the GO terms for biological process, molecular function, and cellular component [25, 26], the Uniprot keywords for biological process, molecular function, and cellular component [27], and the terms of the KEGG pathway analysis [28].

**Table 2:** Mouse embryonic lethal strains with pituitary malformations

Gene^a^	Description, function	Pituitary malformation^b^					Penetrance^c^		Other malformations^d^					OMIM ^e^	Description
		**AHD**	**AHH**	**AHA**	**NHD**	**NHA**	**Affected**	**Total**	**Eye**	**CF**	**Br**	**Go**	**He**		
**Epigenetic and transcriptional regulators**															
*Arid1b*	AT rich interactive domain 1B (SWI-like)	x					5	7	x	x	x	x	x	614556	Coffin-Siris syndrome 1
*Arid2*	AT rich interactive domain 2 (ARID, RFX-like)	x					3	3	na					609539	Coffin-Siris syndrome 6
*Asxl1*	ASXL transcriptional regulator 1	x				x	3	3	na					612990	Bohring-Opitz syndrome, Myelodysplastic syndrome
*Brd2*	Bromodomain containing 2	x			x		5	5	x	x	x	x	x	601540	None reported
*Chtop*	Chromatin target of PRMT1	x			x		4	4	x	x	x	x	x	614206	None reported
*Hira*	Histone cell cycle regulator	x					2	3	na					600237	None reported
*Jarid2*	Jumonji, AT rich interactive domain 2	x					3	3	na					601594	Microcephaly
*Kat14*	Lysine acetyltransferase 14,likely a histone lysine demethylase	x					3	3	na					617501	Global developmental delay, Seizures
*Kdm8*	Lysine (K)-specific demethylase 8	x					3	3	na					610881	Intellectual disability
*Kmt5b*	Lysine methyltransferase 5B	x					3	3	na					610881	Intellectual developmental disorder, autosomal dominant 51
*Mbtd1*	Mbt domain containing 1	x					3	3	na					618705	None reported
*Npat*	Nuclear protein, co-activator of histone transcription	x					1	1	na					601448	None reported
*Setd5*	SET domain containing 5, likely a histone-lysine N-methyltransferase	x					1	4	x				x	615743	Intellectual developmental disorder, autosomal dominant 23
*Tcf7l2*	Transcription factor 7 like 2, T cell specific, HMG box; responsive to WNT signaling	x			x		5	5			x	x	x	602228	Susceptibility to diabetes mellitus, type 2
**RNA regulation and translation**															
*Cnot1*	CCR4-NOT transcription complex, subunit 1	x			x		2	6	x	x	x	x	x	604917	Holoprosencephaly 12, with or without pancreatic agenesis; Vissers-Bodmer syndrome
*Dhx35*	DEAH (Asp-Glu-Ala-His) box polypeptide 35, putative ATP-dependent RNA helicase	x			x		1	1	x	x	x		x	none	None reported
*Nsun2*	NOL1/NOP2/Sun domain family member 2, RNA methyltransferase	x					4	6	x		x	x	x	610916	Intellectual disability
*Prrc2b*	Proline-rich coiled-coil 2B, enables RNA binding activity	x					1	9	x		x		x	619544	None reported
*Smg9*	SMG9 nonsense-mediated mRNA decay factor	x			x		4	6	x	x			x	613176	Heart, brain, ocular malformation; Neurodevelopmental disorder, tremor, pyramidal signs, dyspraxia
*Tent5c*	Terminal nucleotidyltransferase 5C, enables RNA adenylyltransferase activity,mRNA stability	x					5	9		x	x	x	x	613952	None reported
**Protein processing, trafficking, regulation, metabolism**															
*Adamts3*	Disintegrin-like metallopeptidase with thrombospondin type 1 motif, 3						1	7	na					605011	Hennekam lymphangiectasia-lymphedema syndrome 3
*Aldh18a*	Aldehyde dehydrogenase 18 family, member A1	x					1	3	na					138250	Cutis laxa, Spastic paraplegia
*Atg16l1*	Autophagy-related 16-like 1 (S. cerevisiae)	x					2	5	na					610767	Inflammatory bowel disease (Crohn’s disease) 10
*Dph2*	DPH2 homolog, diphthamide biosynthesis—modifies histone for RNA transcription elongation	x					3	3	na					603456	Developmental delay with short stature, dysmorphic facial features, and sparse hair 2
*Ezr*	Ezrin, binds plasma membrane and cytoskeleton	x					1	3	na					123900	None reported
*Gldc*	Glycine decarboxylase	x			x		3	3	na					238300	Glycine encephalopathy
*Kifbp*	Kinesin family binding protein, localizes to mitochondria, maybe involved in transport	x					1	3	x	x	x	x	x	609367	Goldberg-Shprintzen megacolon syndrome
*Marchf5*	Membrane associated ring-CH-type finger 5	x					2	3	na					610637	None reported
*Morc2a*	Microrchidia 2A	x		x			4	14	x	x	x	x	x	616661	Charcot–Marie–Tooth disease
*Nxn*	Nucleoredoxin, redox-dependent regulator of WNT signaling	x					2	3		x		x	x	612895	Robinow syndrome
*Prkab1*	Protein kinase, AMP-activated, beta 1 non-catalytic subunit	x					3	3	na					602740	None reported
*Psat1*	Phosphoserine aminotransferase 1	x					3	3	na					610936	Phosphoserine aminotransferase deficiency, Neu-Laxova syndrome
*Psph*	Phosphoserine phosphatase	x			x	x	7	8	x	x	x	x	x	172480	Intellectual disability; Seizures; Deficiency of phosphoserine phosphatase
*Rundc1*	RUN domain containing 1, may negatively regulate p53 transcriptional activity	x					2	7	x					619250	None reported
*Sh3pxd2a*	SH3 and PX domains 2A, likely a superoxide-generating NADPH oxidase activator	x					10	11	x		x	x	x	619455	None reported
*Ssr2*	Signal sequence receptor, beta; protein translocation across the ER membrane	x					3	3	x		x	x	x	600867	None reported
*Traf2*	TNF receptor-associated factor 2	x					6	6	na					601895	None reported
*Traf6*	TNF receptor-associated factor 6	x					4	9	x		x	x	x	602355	None reported
*Usp3*	Ubiquitin-specific peptidase 3	x					1	3	na					604728	None reported
**Cell signaling, cilia, transmembrane, and extracellular matrix proteins**															
*Atp11a*	ATPase, class VI, type 11A	x					4	4	na					605868	Leukodystrophy, hypomyelinating, 24; Deafness, autosomal dominant 84
*B9d2*	B9 protein domain 2	x		x	x		4	4	na					611951	Meckel syndrome 10; Joubert syndrome 34
*Cc2d2a*	Coiled-coil and C2 domain containing 2A	x	x		x	x	6	10	x	x	x	x	x	612013	COACH syndrome, Joubert Syndrome type 9; Meckel Syndrome type 6
*Ehd1*	EH-domain containing 1, vesicle trafficking, receptor recycling, cilia	x					2	3	na					605888	None reported
*Grin1*	Glutamate receptor, ionotropic, NMDA1 (zeta 1)	x					1	3	na					138249	Developmental and epileptic encephalopathy 101; Neurodevelopmental disorder, seizure
*Mks1*	MKS transition zone complex subunit 1	x	x			x	2	2	na					609883	Bardet-Biedl syndrome; Joubert syndrome; Meckel syndrome
*Plxnb2*	Plexin B2, transmembrane, axon guidance	x			x		1	3	na					604293	None reported
*Rala*	V-ral simian leukemia viral oncogene A (ras related), transmembrane signaling, mitochondrial fission	x					6	8	na					179550	Hiatt-Neu-Cooper neurodevelopmental syndrome
*Rpgrip1l*	Rpgrip1-like, localizes to basal body centrosome, cilia	x				x	6	6	x	x	x		x	610937	Joubert Syndrome. Meckel Gruber Syndrome
*Sptbn1*	Spectrin beta, non-erythrocytic 1, actin binding transmembrane protein	x					2	3	na					182790	Developmental delay, impaired speech, and behavioral abnormalities
*Vangl1*	VANGL planar cell polarity 1	x					3	3	na					610132	Caudal regression syndrome; susceptibility to neural tube defects
**Other/unknown**															
*Mad2l2*	MAD2 mitotic arrest deficient-like 2	x					2	2	na					604094	Fanconi anemia, complementation group V

^a^Hyperlink to gene cards

^b^Type of malformation: *AHD*, adenohypophysis dysmorphology; *AHH*, adenohypophysis hypoplasia; *AHA*, adenohypophysis absent; *NHD*, neurohypophysis dysmorphology; *NHA*, neurohypophysis absent

^c^# embryos affected/total homozygotes characterized

^d^Other malformations: eye, may include optic cup, stalk, eye muscle, lens, optic chiasm, etc.; *CF*, craniofacial, may include cleft palate, dental defects, facial bones; *Br*, brain, CNS; *Go*, gonad, may include ovary, testis, Wolffian duct, mullerian duct; *He*, heart

^e^Hyperlink to gene in OMIM

### Mice

Wild-type C56BL/6 J and Tg(RARE-Hspa1b/lacZ)12Jrt/J (Jackson Laboratory strains 000664 and 008477) mice used for this study were approved by the University of Michigan Institutional Animal Care and Use Committee and the Animal Care and Use Office (protocol #PRO00010454, expiration date 9/28/2024). Immunofluorescence experiments using mouse embryonic samples were approved by the Institutional Animal Care and Use Committees of Southern Illinois University (protocol #22–026, approved 7/29/2022) and the University of South Carolina (protocol #2576–101677-122221, approved 12/22/2021). Animals were housed in 12-h light:12-h dark cycles, and food and water were provided ad libitum. For embryonic time points, one male and one female mouse were paired, and the female mouse was checked for the presence of a copulation plug the following morning. The day a copulation plug was observed was designated as embryonic day 0.5 (e0.5). Pregnant mice were euthanized using carbon dioxide asphyxiation, and embryos were collected at appropriate time points and fixed in 4% formaldehyde in phosphate-buffered saline.

### Immunohistochemistry

Fixed embryos were dehydrated, permeabilized, and embedded in paraffin for sectioning at 6 µm [29]. Select paraffin sections were processed for immunofluorescence using an antibody for EZR (Abcam, cat# ab4069) or SSR2 (Fisher Scientific, cat# 50–172-6174) diluted 1:100 in 1% blocking reagent (Life Technologies), followed by biotin-conjugated donkey anti-rabbit secondary (Life Technologies, cat# A16033) diluted 1:100 in 1% blocking reagent, and detected with streptavidin-conjugated DyLight 488 (Thermo Scientific, cat# 21832) for EZR or streptavidin-conjugated Alexa 488 (Jackson ImmunoResearch, cat# 016–540-084) for SSR2 diluted 1:100 in 1% blocking reagent. Sections were counterstained with 300 nM 4′,6-diamidino-2-phenylindole (DAPI) before mounting in fluorescence mounting media.

### Single-cell RNA sequencing

Rathke’s pouches were harvested from e12.5 and e14.5 embryos. One e12.5 pituitary gland from a retinoic acid reporter mouse (RARE-LacZ JAX strain #008477) was dissociated in an enzyme mix containing 0.5% w/v collagenase type 2 (Lorne Laboratories, Reading, UK), 0.1 × trypsin (Invitrogen, Carlsbad, CA), 50 μg/mL DNase I (Worthington Biochemical, Lakewood, NJ), and 2.5 μg/mL amphotericin B (Fungizone; Invitrogen) in Hanks balanced salt solution (Invitrogen) for 1 h at 37 °C. The C57BL/6 J e14.5 sample was dissociated into single cells in 50 µl of a solution containing 1 mg/ml papain (Roche, cat# 10,108,014,001), 5 mM L-cysteine, 1 mM EDTA, and 0.6 mM 2-mercaptoethanol. Dissociation was performed at 37 °C for 5 min, with titration every 2 min. The samples were then neutralized in 75 µl of neurobasal media (Invitrogen, cat# 12,348,017) with 10% FBS (Corning, cat# MT35010CV). The single cells were treated with 0.75 µl of RNase-free Recombinant DNase I (Roche, cat#4,716,728,001) at 37 °C for 5 min. Single cells were then pelleted in a pre-chilled microfuge for 5 min at 300 relative centrifugal force (RCF). The supernatant was aspirated, and the cells were resuspended in 50 µl neurobasal media with 1% FBS. Cell viability was analyzed using the Countess 3 cell counter (Invitrogen). Samples were 3′ end sequenced using the 10 × genomics platform (v2 for e12.5, v3 for e14.5 samples) by the Advanced Genomics Core at the University of Michigan. Sequences are deposited in the Gene Expression Omnibus under series accession number GSE246211 [30], e12.5 sample GSM7864906 [31], and e14.5 sample GSM7864907 [32]. For the e12.5 sample, 1701 cells were sequenced, and the mean reads per cell was 121,698. For the e14.5 samples, 19,625 cells were sequenced, and the mean reads per cell was 27,387. Additional File 2: Fig. S1 shows the feature, count, and percent mitochondrial data before and after normalizing for nFeature_RNA > 500, nfeatures > 200 and < 2000, and percent mitochondrial DNA < 15%. Sequencing reads were processed, and cells were clustered using Seurat 4.1.0. The pituitary cluster is defined by the pituitary transcription factors *Lhx3*, *Pitx1*, *Pitx2*, *Pou1f1*, *Prop1*, and *Pomc* and the neural cluster is defined by the transcription factors *Rax*, *Nkx2.1*, and *Nkx2.4*, as well as *Fgf8* and *Fgf10.* The vasculature profile includes *Pecam1*, *Hba-x*, *Rgs5*, *Igfbp7*, *Ramp2*, and *C1qc*, while the mesenchyme expresses *Penk*, *Lgals1*, *Dan*, and* Foxd1.*

### Informed consent and patient recruitment

The study was approved by the ethical committee of the Hospital Garrahan, Buenos Aires, Argentina. Informed consent forms were signed by either adult individuals with CH or the parents/guardians of the children with CH. Patients were diagnosed with hormone deficiencies according to previously described and internationally accepted criteria by the Endocrine divisions of the pediatric hospitals in Argentina [1, 33]. The whole cohort study was published in [34]. In brief, the cohort included 143 individuals, corresponding to 137 cases: 131 cases were sporadic, and 6 cases were familial, including a total of 86 males and 57 females. Two patients from that study (patient 54 and patient 55) are reported here. Genomic DNA was isolated from peripheral blood and used for whole exome sequencing.

### Human whole exome sequencing

Whole exome sequencing (WES) of the patients was performed as part of the “End the Diagnostic Odyssey Grant” financed by 3Billion, Seoul, Korea [34]. Exome capture was performed using xGen Exome Research Panel v2 (Integrated DNA Technologies, Coralville, IA, USA) and sequencing was performed using NovaSeq 6000 (Illumina, San Diego, CA, USA). Raw reads were aligned to GRCh38 using the Barrow Wheelers Aligner (BWA) [35] and processed according to the recommendations of the Genome Analysis Toolkit (GATK) [36, 37]. Duplicated reads were discarded using PICARD and haplotyping, genotyping, and variant calling were performed using Haplotype Caller. The VCF was then annotated using dbSNP, ExAC, GnomAD, ClinVar, Polyphen, SIFT, Mutation Taster, and ACMG [38–44]. Annotated VCF files were uploaded to Bitgenia’s platform (https://www.bitgenia.com/b-platform/). Genetic variants were screened using the panel of 51 candidate genes and filtered by frequency (> 0.01 in GnomAD).

### Sanger sequencing

To confirm variants in *MORC2* primers were designed using Primer-BLAST [45]. PCR products were amplified in 50 µl reactions, using Pfu DNA polymerase (Inbio Highway, #K1100). PCR products were gel purified using Zymoclean^Tm^ Gel DNA Recovery Kit (Zymo, cat#D4007) and sent for Sanger Sequencing (Macrogen).

### Conservation analysis and structural modeling

Conservation analysis was performed using Clustal Omega with default parameters [46]. Uniprot structures used were as follows: 1 Q9Y6X9 (*Homo sapiens*), K7D393 (*Pan troglodytes*), Q69ZX6 (*Mus musculus*), A0A4X1V6A2 (*Sus scrofa*), E1BGP4 (*Bos Taurus*), A0A8I3PH66 (*Canis lupus*), M3W1K4 (*Felis catus*), G3T998 (*Loxodonta africana*), and A0A455AZN6 (*Physeter macrocephalus*) for MORC2. Results were visualized with Jalview [47]. Structural changes in MORC2 were performed on the 5OF9 crystal [48]. The mutations were introduced and modeled using Modeller [49] and visualized on VMD [50].

## Results

### Identification of lethal and sub-viable mice with pituitary malformations

The Wellcome Trust Sanger Institute Mouse Genetics project pipeline [46] used HREM to characterize embryonic morphology in 209 knockout mice with embryonic lethal or sub-viable phenotypes, defined as strains lacking viable homozygotes at postnatal day 14 (p14) and/or p21 [17, 18]. The high-resolution images allowed us to visually screen sagittal sections for pituitary malformations evident at e14.5 for all 209 knockout lines, from which we identified 51 that have pituitary malformations (Table 2). Only one of these genes, *Tcf7l2*, has a known role in mouse pituitary gland organogenesis [19, 51]. As HREM data was only available from one embryonic time point, our analysis is inherently limited. The absence of a pituitary malformation in the other knockout mice examined in the pipeline does not eliminate these genes from consideration in pituitary organogenesis studies as it is possible that a pituitary malformation or specification defect may be present later in embryogenesis.

The penetrance of the pituitary gland phenotype was variable (Table 2). On average, 4.7 homozygous mutants were analyzed for each gene, and the range was 1–14. In 47 cases where at least 3 mutant embryos could be examined, 40% were fully penetrant, 34% were strongly penetrant (≥ 50% penetrance), and 26% were weakly penetrant (< 50% penetrance). The variability in penetrance for pituitary malformations is in line with the phenotypic variability observed more broadly in the DMDD database, despite the use of the inbred mouse strain C57BL/6N for the generation of the knockout mice [52].

CH often presents with other congenital malformations, including abnormalities in the eyes (26%), brain (26%), gonads (18%), and craniofacial regions (21%) [1]. The DMDD had officially annotated 21 of the 51 genes with pituitary malformations. All these annotated cases had a malformation in at least one other tissue: heart (95%), eye (86%), brain (81%), gonad (71%), or craniofacial region (62%) (Table 2), and 38% had defects in all five tissues (the full list of observed malformations is included in Additional File 3: Table S2). The high incidence of heart defects likely contributes to the poor viability. These data indicate that the recessive embryonic lethal mouse strains exhibit complex structural defects that affect the same tissues as human patients with CH, but the penetrance of effects on other organs is higher in the knockout mice.

We hypothesized that these 51 genes with pituitary malformations in mice would be excellent candidate genes for CH in humans. As a first step toward understanding their role in pituitary gland organogenesis, we queried each candidate gene in the OMIM database for associations with human disease (Table 2). Thirty-two candidate genes are associated with human disease (63%), only one of which, *Arid1b*, was previously associated with HPE, SOD, or CH (Table 2).

### Pathway analysis of CH genes and candidates from DMDD

Other than *Tcf7l2*, the genes mutated in these embryonic lethal or sub-viable mice do not have defined molecular functions during pituitary gland organogenesis. We grouped candidate genes and CH-causing genes into broad functional categories listed in Tables 1 and 2, based on the molecular function associated with their gene name. To analyze the molecular pathways represented by these candidate genes further, we used DAVID to identify GO terms, Kyoto Encyclopedia of Genes and Genomes (KEGG) pathway, and Uniprot key words that are associated with our list of candidate genes and the list of known CH genes (Additional File 1: Table S1) [23, 24]. These terms were analyzed to identify those overrepresented in each list and then clustered to identify functionally related annotation terms. The annotation clusters with a group enrichment score of greater than 1.3, representing a *p* < 0.05, are presented in Table 3. The annotation cluster description is a specific GO term or Uniprot key word selected to represent all the annotations in the cluster. The full list of annotated terms in each cluster is presented in Additional File 4: Table S3.

**Table 3:** DAVID functional annotation clustering of congenital hypopituitarism genes and candidate genes

Annotation cluster	Cluster description	Enrichment score	Genes
**Known CH genes**			
1	Transcription regulation	8.93	ARID1B, ARNT2, BLM, CHD7, FOXA2, FOXL2, GLI2, GLI3, GLI4, HESX1, HMGA2, HNF1A, HNRNPU, LHX3, LHX4, NFKB2, NKX2-1, NONO, OTX2, PAX6, POU1F1, PROP1, RAX, SIX1, SIX5, SIX6, SMCHD1, SOX2, SOX3, TCF7L1, TGIF1, ZIC2
2	Dorsal/ventral pattern formation	4.32	BMP2, BMP4, FGF8, GLI3, HHIP, IFT172, PROP1, SHH
3	Hedgehog signaling pathway	3.78	BMP2, CDON, GLI2, GLI3, GPR161, HHIP, IFT172, KCNQ1, PAX6, SHH, TBC1D32, WDR11
4	Branching involved in ureteric bud morphogenesis	3.22	ARNT2, BMP2, BMP4, CDON, FGFR1, GLI2, GLI3, HHIP, HNRNPU, PAX6, POU1F1, SHH, SOX2, TBC1D32, TCF7L1
5	Cilium	2.92	CDON, GLI2, GLI3, GPR161, IFT172, L1CAM, PAX6, ROBO1, SHH, TBC1D32, TMEM67, WDR11, TBC1D32, TCF7L1
6	Telencephalon regionalization	2.28	ANOS1, ARNT2, BMP2, BMP4, FGF8, FGFR1, HHIP, IGSF1, IGSF10, LAMB2, PCSK1, SEMA3A, SHH, SPINK5
7	Neurogenesis	1.32	ARID1B, L1CAM, ROBO1, SEMA3A, SEMA3A, SHH, ZIC2, ZSWIM6
**Candidate genes**			
1	Chromatin binding	3.3	ARID1B, ARID2, ASXL1, BRD2, HIRA, JARID2, KDM8, KMT5B, MBTD1, SETD5, USP3
2	Transcription regulation	3.13	ASXL1, B9D2, BRD2, CHTOP, CNOT1, HIRA, JARID2, KAT14, KDM8, KMT5B, MAD2L2, MBTD1, MORC2, NPAT, NSUN2, NXN, PRKAB1, SETD5, SPTBN1, TCF7L2, TENT5C, TRAF6, USP3
3	Cilium	1.98	B9D2, CC2D2A, EHD1, EZR, MKS1, RPGRIP1L, SH3PXD2A
4	Ciliary transition zone	1.88	B9D2, CC2D2A, EZR, KIFBP, MAD2L2, MKS1, NSUN2, RPGRIP1L, SPTBN1, TENT5C
5	Amino acid biosynthesis	1.48	ALDH18A1, GLDC, KMT5B, PSAT1, PSPH
6	Glycine, serine, and threonine metabolism	1.34	ALDH18A1, GLDC, KMT5B, PSAT1, PSPH

The enriched annotated clusters for the list of known CH genes and candidate genes are similar in that they both contain numerous genes involved in transcriptional regulation. However, the candidate genes are also enriched for chromatin binding, which likely represents epigenetic transcriptional regulation, a largely unexplored cause for CH. The known CH genes are enriched in genes involved in hedgehog signaling, which utilizes the primary cilia as a signaling center [53]; therefore, annotation terms associated with the cilia are also enriched among the known CH genes. While the candidate gene list is not enriched in genes associated with hedgehog signaling, it is enriched in genes associated with the ciliary transition zone. Therefore, the candidate gene list extends the list of cilia-associated genes to more structural ones, which have not been previously linked to CH.

The candidate genes also include two novel enriched clusters related to amino acid metabolism, specifically glycine and serine. Two genes that encode for the enzymes, PSAT1 and PSPH, which are necessary for L-serine synthesis, and the gene for GLDC, which is an enzyme in the glycine cleavage system, are included in these metabolic clusters [54].

### Morphological analysis of the pituitary malformations

The pituitary malformations observed in the DMDD HREM data include absent, hypoplastic, and dysmorphic anterior and posterior lobes. Embryos with an absent or small anterior lobe may arise from a deficiency in signaling between the ventral diencephalon and the oral ectoderm. Within the candidate genes, many had a phenotype suggestive of a reduction in ventral diencephalon paracrine signaling. At e14.5, a wild-type pituitary gland has a bulbous infundibulum dorsal to the anterior and intermediate lobes, which are separated by a sigmoidal cleft (Fig. 1A and Additional File 5: Fig S2A and Additional File 6: Fig S3A). *Rpgrip1l*^*−/−*^ embryos have an absent pituitary (Fig. 1B), while *Mks1*^*−/−*^, *Cc2d2a*^*−/−*^, and *B9d2*^*−/−*^ have small anterior lobes (Fig. 1 C and D, and Additional File 5: Fig S2B). Interestingly, these candidate genes all function in cilia formation, suggesting the hypoplastic phenotype may result from a ciliary deficiency. Phenotypic variability can occur between embryos of the same genotype amongst the candidate genes; for instance, one *Cc2d2a*^*−/−*^ embryo appears to have an initial induction of pituitary progenitors followed by a failure in proliferation and/or survival (Additional File 5: Fig S2C), while other *Cc2d2a*^*−/−*^ embryos have variable hypoplastic anterior lobes (Fig. 1D and Additional File 5: Fig S2D and S2E).

Pituitary malformations can also result from an expansion of the signaling center in the ventral diencephalon, which causes a broader region of the oral ectoderm to be induced to form pituitary progenitors. This is the case with *Tcf7l2*^*−/−*^, and the malformation observed in the DMDD data is similar to previously characterized malformations with broader induction of pituitary progenitors (Fig. 1E) [19]. *Gldc*^*−/−*^**, ***Psat1*^*−/−*^*,* and *Psph*^*−/−*^ embryos have an expanded anterior lobe that fails to separate from the oral ectoderm (Fig. 1F–H). *Gldc*, *Psat1*, and *Psph* all function in amino acid metabolism, pointing to a possible common mechanism for these genes involved in pituitary gland organogenesis. *Asxl1* is an epigenetic regulator that results in a similar expansion of the pituitary progenitors (Additional File 5: Fig S2F).

Chromatin organization by epigenetic regulators, like *Asxl1*, broadly impacts gene expression, and loss of those transcriptional regulators can generate diverse phenotypes as a result. A diversity of phenotypes is observed among the 15 candidate genes that are classified as epigenetic or transcriptional regulators. In contrast to the dysmorphology of *Asxl1*^*−/−*^ pituitaries, the pituitaries of the following strains are hypoplastic: *Morc2a*^*−/−*^, *Mbdtd*^*−/−*^, *Brd2*^*−/−*^, *Npat*^*−/−*^, and *Jarid2*^*−/−*^ (Fig. 1I and Additional File 5: Fig S2G – S2J). *Arid1b*^*−/−*^, *Kat14*^*−/−*^ and *Kdm8*^*−/−*^ embryos do not have hypoplastic anterior lobes but have an altered appearance of the cleft (Fig. 1J–L). For *Arid1b*^*−/−*^ and *Kat14*^*−/−*^, the intermediate lobe is more prominently affected, with epithelial cells extending to the dorsal side of the posterior lobe. In *Kdm8*^*−/−*^ embryos the cleft appears “branchy” with a more prominent effect on the anterior lobe side. Homozygous null embryos for *Mad2l2*, *Cnot1*, and *Hira*^*+/-*^ embryos have dysmorphic clefts similar to *Kat14*^*−/−*^ where the epithelial cells extend to the dorsal side of the posterior lobe (Additional File 5: Fig S2K–S2M), while the epigenetic and transcriptional regulators *Kmt5b*^*−/−*^, and *Chtop*^*−/−*^ are more similar to *Kdm8*^*−/−*^ with an anterior lobe dysmorphology (Additional File 5: Fig S2N and S2O). Note that the *Hira*^*+/-*^ phenotype is observed in heterozygous embryos. *Hira*^*−/−*^ are embryonic lethal by e11 resulting from gastrulation and placentation defects [55].

The genes discussed so far were presented first because of their association with GO terms enriched in our list of candidate genes. There are numerous genes with pituitary dysmorphologies that are not associated with cilia, glycine and serine metabolism, or epigenetics and transcriptional regulation. A small anterior lobe is observed for *Atg16l1*^*−/−*^, and *Smg9*^*−/−*^ has a dysmorphic posterior lobe (Additional File 6: Fig S3B and S3C). *Atp11a*^*−/−*^, *Arid2*^*−/−*^*, Dph2*^*−/−*^, *Edh1*^*−/−*^, *Grin1*^*−/−*^, *Plxnb2*^*−/−*^, and *Usp*^*−/−*^ have extensions of the intermediate lobe into the cleft that approach the anterior lobe (Additional File 6: Fig S3C—S3J). *Adamts3*^*−/−*^ and *Prkab1*^*−/−*^ have intermediate lobes that extend to the dorsal side of the posterior lobe (Additional File 6: Fig S3K and S3L)*. Aldh18a*^*−/−*^ and *Dhx35*^*−/−*^ have dysmorphic clefts where the anterior lobe is more affected (Additional File 6: Fig S3M and S3N). *Kifbp*^*−/−*^, *Rala*^*−/−*^, *Rundc*^*−/−*^, *Sptbn1*^*−/−*^, *Tent5c*^*−/−*^*,* and *Vangl*^*−/−*^ have branchy clefts (Additional File 6: Fig S3O– S3T), while *Nsun2*^*−/−*^ has a branchy cleft combined with a hypoplastic adenohypophysis (Additional File 6: Fig S3U). *Nxn*^*−/−*^ and *Prrc2b*^*−/−*^ embryos appear to have anterior lobe tissue that protrudes into the cleft (Additional File 6: Fig S3V and S3W).

### Expression of genes that cause pituitary malformations

**Fig. 2:**
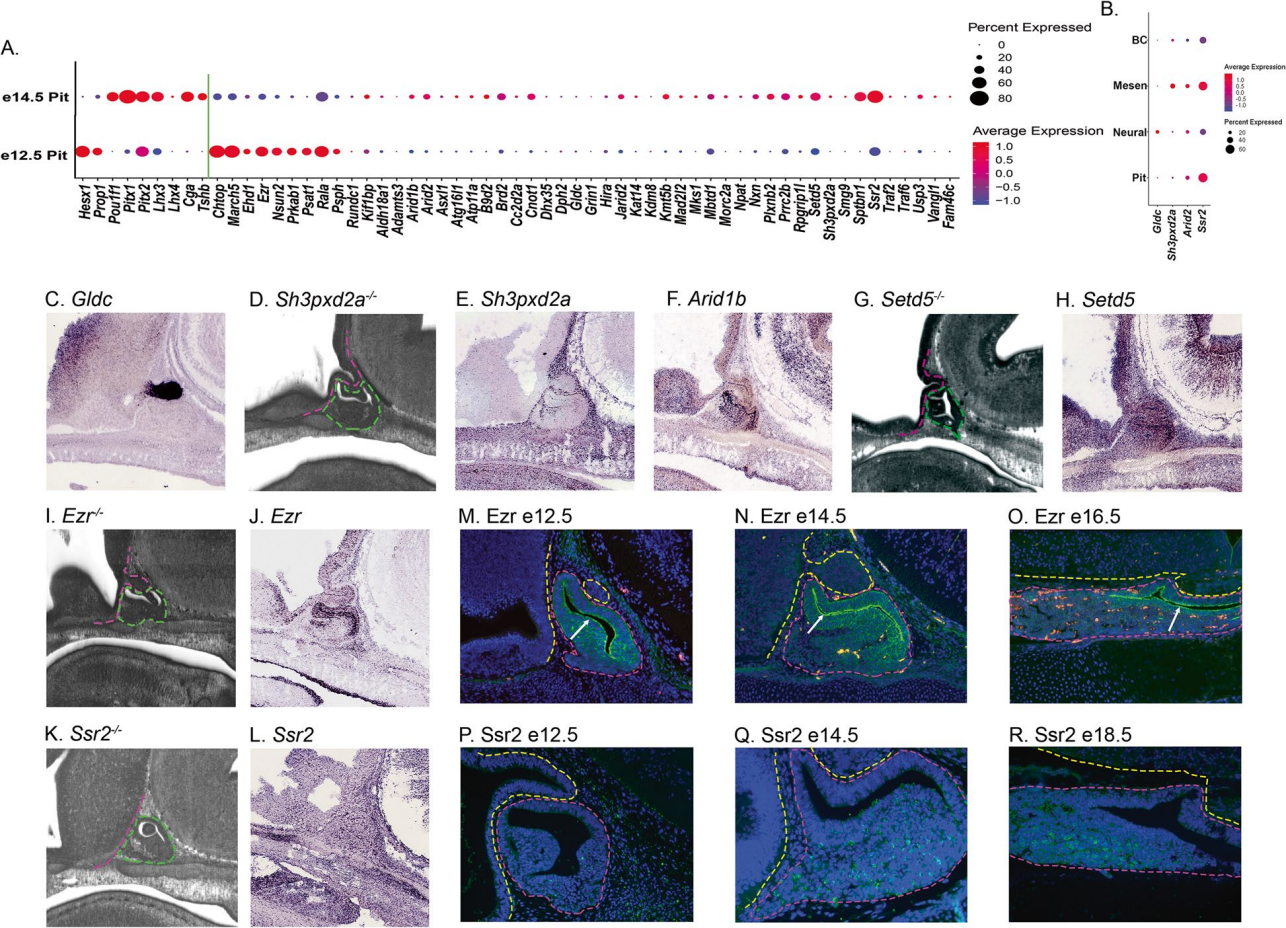
Expression analysis of the CH candidate genes. **A** scRNA-seq dot plots for all candidate genes (to the right of the green line) from dissected e12.5 and e14.5 pituitary glands. Note that *Tent5c* uses the alternate gene symbol *Fam46c* in this analysis. Dot size represents the percent of cells in each condition that express the candidate gene, while the color represents the relative expression level in those cells. Nine genes (*Hesx1* to *Tshb* to the left of the green line) with known expression in the developing pituitary gland are included for qualitative comparison. **B** Dot plots for expression in cells sorted into mesenchyme (Mesen), endothelial cells (BC), ventral diencephalon (Neural), and pituitary anterior lobe (Pit) at e14.5 for select candidate CH genes. Dot size and color are the same as in **A**. **C** GenePaint RNA in situ hybridization image for *Gldc.***D** DMDD HREM stackviewer image for *Sh3pxd2a*^*−/−*^. **E** and **F** GenePaint RNA in situ hybridization images for *Sh3pxd2a* and *Arid1b*. **G** DMDD HREM stackviewer image for *Setd5*^*−/−−*^. **H** GenePaint RNA in situ hybridization image for *Setd5*^*−/−*^. **I** DMDD HREM stackviewer image for *Ezr*^*−/−*^. **J** GenePaint RNA in situ hybridization image for *Ezr*. **K** DMDD HREM stackviewer image for *Ssr2*.^*−/−*^. **L** GenePaint RNA in situ hybridization image for *Ssr2.***M**–**O** Immunofluorescence images for Ezr at e12.5 (**M**), e14.5 (**N**), and e18.5 (**O**). **P**–**R** Immunofluorescence images for Ssr2 at e12.5 (**P**), e14.5 (**Q**), and e18.5 (**R**)

Pituitary malformations evident at e14.5 could be caused by genes expressed in the pituitary or by genes expressed in surrounding tissues that affect the pituitary indirectly. We utilized scRNA-seq from dissected e12.5 and e14.5 wild-type pituitary glands to determine whether the CH candidate genes were expressed within the pituitary gland before or at the time the malformation was noted (Fig. 2A) [30]. Seven transcription factors and two hormone subunits known to be expressed in the pituitary anterior lobe were included in the temporal analysis for qualitative expression level comparison with the candidate genes. All candidate genes were identified in the scRNA-seq analysis. Some genes have a higher expression at e12.5 than at e14.5 (see for example *March5*, *Nsun2*, and *Rala*). Most genes have a lower expression at e12.5 followed by higher expression at e14.5 (see for example *Plxnb2*, *Sptbn1*, and *Ssr2*).

We used the scRNA-seq data from normal mouse pituitary glands at e14.5 to assess the tissue-specific expression of the candidate genes by sorting the data to identify cell populations from the ventral diencephalon (neural), pituitary cells from the anterior lobe, mesenchymal cells adjacent to the pituitary gland, and vasculature and blood cells (see Methods for details). We found that some genes are more highly expressed in specific cell types. *Gldc* has higher expression in neural cells, *Sh3pxd2a* is more highly expressed in the pituitary adjacent mesenchyme (Fig. 2B), *Arid1b* and *Ezr* have higher expression in the anterior lobe, and *Ssr2* is more highly expressed in the anterior lobe and mesenchyme. The expression analysis for the full list of CH candidate genes in each cluster is presented in Additional File 7: Fig S4.

To assess the spatial expression of the candidate genes, we queried the e14.5 RNA in situ hybridization data at GenePaint.org for all candidate genes (Additional File 8: Fig. S5 and Additional File 9: Fig S6). *Gldc*^*−/−*^ embryos have a placodal expansion phenotype, suggestive of an expansion of the signaling center in the ventral diencephalon (Fig. 1E), and the *Gldc* RNA in situ hybridization shows striking expression in the posterior lobe (Fig. 2C). The *Sh3pxd2a*^*−/−*^ embryos have a malformed intermediate lobe, including a small extension into the cleft and an interrupted sphenoid bone (Fig. 2D). *Sh3pxd2a* RNA in situ hybridization expression confirms higher level expression in the pituitary adjacent mesenchyme (Fig. 2E), suggesting that interactions between the mesenchyme and the pituitary progenitors are necessary for pituitary gland organogenesis. *Arid1b*^*−/−*^ embryos have an expansion of the intermediate lobe dorsal to the posterior lobe (Fig. 1J) and *Arid1b* RNA in situ hybridization shows more intense staining around the pituitary cleft (Fig. 2F). *Setd5*^*−/−*^ embryos have hypoplastic posterior and anterior lobes with a branchy cleft (Fig. 2G). *Setd5* mRNA is strongly expressed in both the ventral diencephalon and the pituitary intermediate and anterior lobes (Fig. 2H). *Ezr*^*−/−*^ embryos also have a protrusion of the intermediate lobe into the cleft (Fig. 2I). *Ezr* is more strongly expressed in the proliferative zone for pituitary progenitor cells (Fig. 2J). Ss*r2*^*−/−*^ embryos have a protrusion of anterior lobe tissue into the cleft (Fig. 2K), while *Ssr2* RNA expression by in situ hybridization is observed in all tissues (Fig. 2L).

We extended our expression analysis for *Ezr* and *Ssr2* by using available antibodies for immunofluorescence experiments. EZR links the plasma membrane with the F-actin cortical network and is enriched at the apical side of polarized epithelia [56]. Immunofluorescence on wild-type mice using an EZR antibody shows stronger expression on the apical side of cells lining the cleft at e12.5, e14.5, and e18.5, which is characteristic of polarized epithelial cells (Fig. 2M–O). Cells that are not adjacent to the lumen do not have a polarized expression of EZR; instead, it is observed in the cytosolic region throughout the cell. By e18.5, EZR expression is decreased throughout the anterior lobe. *Ssr2* encodes a component of the signal sequence recognition particle. Low levels of *Ssr2* are detected at e12.5 by scRNA-seq, and very little SSR2 protein is observed at e12.5. At e14.5, increasing amounts of SSR2 are observed in individual cells, a pattern that is maintained at e18.5 (Fig. 2P–R). It is interesting that the protein expression seems to be limited to a subset of anterior lobe cells at e18.5, located in the pituitary parenchyma and away from the stem cell niche, despite the broader RNA expression observed by RNA in situ hybridization. Taken together the expression data demonstrates that the DMDD genes are expressed in and around the developing pituitary gland, strengthening their status as candidate genes for potentially causing CH.

### Identification of DMDD candidate gene variants in children with CH

**Fig. 3:**
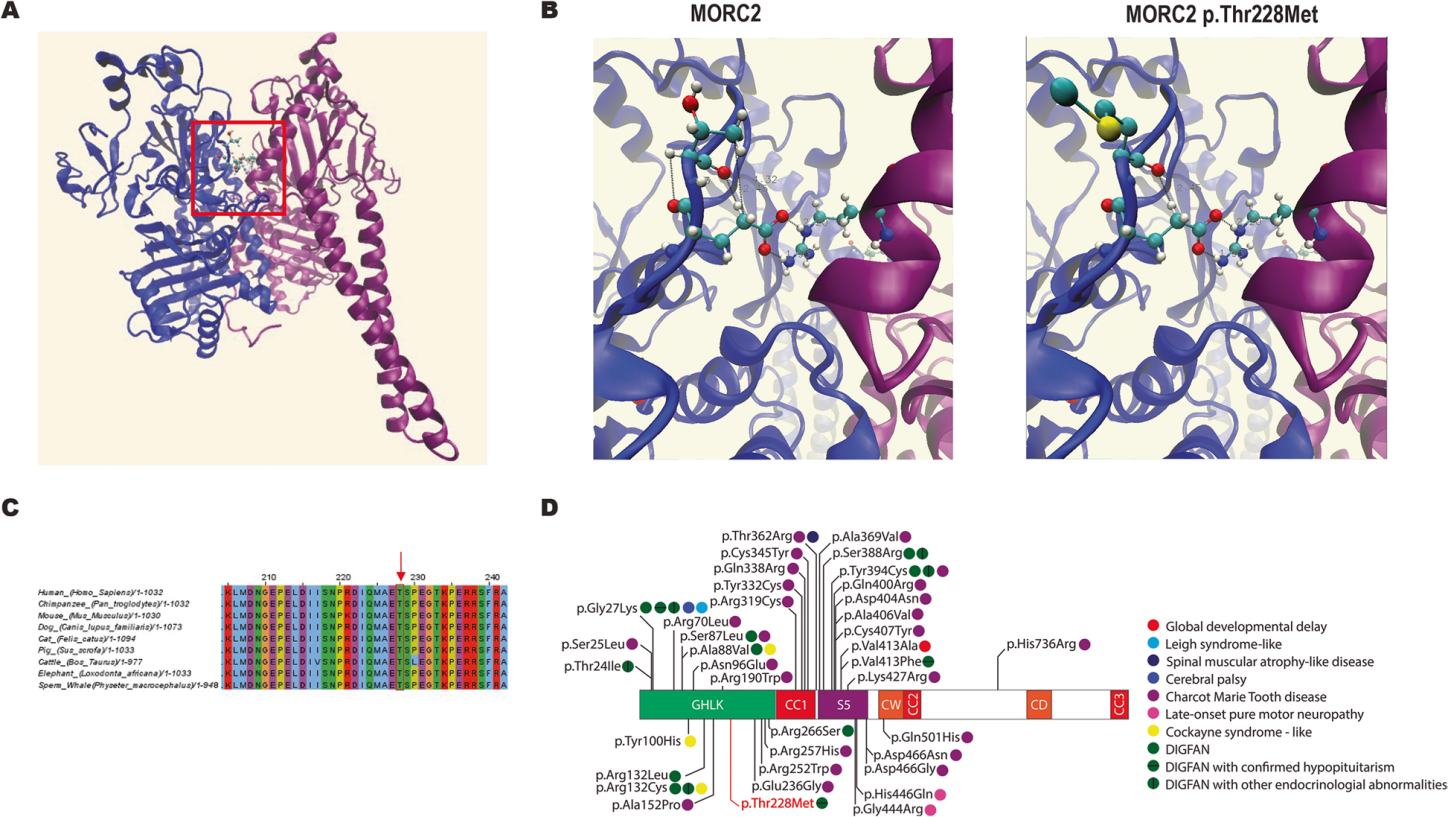
Heterozygous DNA sequence variant in *MORC2* identified in a patient with CPHD. **A** Predicted protein structure of a MORC2 dimer, with the monomers shown in blue and purple. The boxed region indicates the interaction domain that contains Thr 228. **B** Close-up view of the boxed region in **A** showing the interactions between Thr 228 and Gly 227 and between Thr 228 and Arg 437 (left) and how those bonds are disrupted in MORC2 p.Thr228Met (right). **C** Protein alignment of MORC2 with select mammalian species. The red box indicates Thr 228, which is conserved across mammals. **D** Schematic of MORC2, showing the domain structure and location of known heterozygous variants and the associated phenotypes

We conducted whole exome sequencing of 137 children with hypopituitarism [34], and we reported two cases with variants in *MORC2* and *SETD5*, the human orthologs of the IMPC candidate gene *Morc2a* and *Setd5*. The first patient carries a heterozygous variant in *MORC2* (ENST00000397641.8): c.683C > T (p.Thr228Met) (rs774960940), which we consider likely pathogenic by four American College of Medical Genetics and Genomics (ACMG) reporting criteria (PM1, PM2, PP2, PP3) [7]. This patient is a girl who was first evaluated at 1.6 years-old due to persistent neonatal hypoglycemia and epilepsy. She showed combined pituitary insufficiency (ACTH, TSH, and GH). Her highest repeated cortisol value at 8:00 AM was 5.3 µg/dL (ref. value 8.7–22.4 µg/dL), her total T3 was 69 ng/dL (reference values 87–178 ng/dL) and her free T4 was 0.36 ng/dL (reference values 0.65–2 ng/dL). After treatment with hydrocortisone and levothyroxine, her GH peak response to the arginine clonidine stimulation test was 0.78 ng/mL (reference value 4.8 ng/mL), and treatment with recombinant human GH was started. Her MRI showed white matter anomalies, ventricular asymmetry, a hypoplastic anterior pituitary and an ectopic posterior pituitary. She is now 12 years old with a height of − 0.64 SDS, a weight of − 0.62 SDS, prepubertal status, severe developmental delay, muscle hypotonia, and significant joint hyperlaxity. Her hypotonia is now extremely severe, to the point where she is wheelchair bound. This is common in patients with *MORC2* variants [57], but extremely rare in cases of congenital hypopituitarism, which further supports the link between variant and phenotype. The variant has a low frequency in GnomAD 4.0 (21 heterozygotes for a frequency of 0.00002, no homozygotes). Some *MORC2* variants cause late-onset neuropathy, so rare individuals with this variant in the GnomAD database may not have presented symptoms yet or the variant might exhibit incomplete penetrance. Both parents are unaffected (DNA was not available). Structural analysis with MutaBind2 showed that threonine 228 is located at the interaction surface of the MORC2 homodimer [58]. Making use of available homodimer crystals, we show that the change of threonine 228 to methionine is likely to destabilize the loop in which the residue is found. Several interactions with the neighboring glutamic acid at position 227 are lost. This residue is conserved across mammals and is in the Histidine kinase-like ATPase domain (Fig. 3C and D). This residue is key for homodimer formation, forming two hydrogen bonds with arginine 437 of the interacting MORC2 protein (Fig. 3). In silico analysis of dimer stability upon mutating the residue was estimated at − 0.149 kcat/mol ΔΔG_Affinity_ according to mCSM-PPI2, meaning decreased affinity and therefore less homodimer formation [44].

**Fig. 4:**
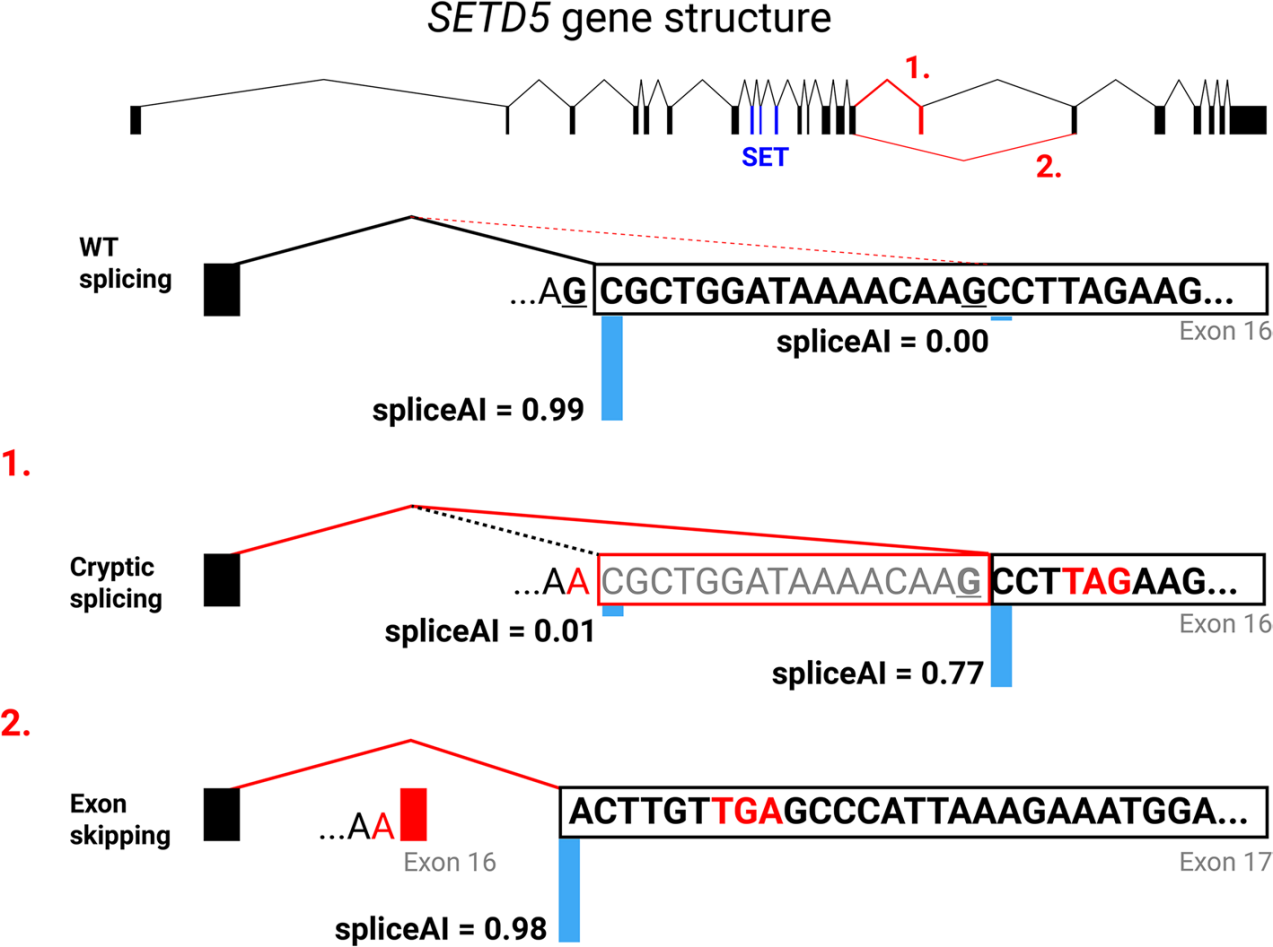
Gene structure of *SETD5* and splicing analysis of the mutant transcript. Exons are shown as black boxes, except for exons 8 to 10 that form the SET domain, depicted in blue, and the affected exon 16, depicted in red. Wildtype splicing events between exons 15 and 16 are shown as black lines. The affected intronic base is highlighted in bold and underlined in the wildtype splicing event. The G to A variant generates two possible splicing events. Cryptic splicing between exon 15 and the cryptic splice site in exon 16 is diagrammed in 1 with the red box indicating the deleted bases. Exon skipping from exon 15 to 17 is diagrammed in 2. The spliceAI score for the wildtype and mutated acceptor site (including the cryptic acceptor site) are shown with an illustrative light blue bar. The resulting premature stop codons and the lost acceptor site in the alternative splicing events are highlighted in red

In a second case, a pathogenic variant in *SETD5* was identified (ENST00000402198.5: c.2347-1G > A, ACMG criteria PVS1, PM2, PP3, PP5). The variant has been previously reported as likely pathogenic in Clinvar (RCV000578558) and in two separate publications, but no clinical information was provided in these instances [59–61]. The variant is absent from GnomAD v4.0 and predicted to affect splicing (spliceAI acceptor loss prediction score = 0.99) (Fig. 4). The loss of the canonical splicing acceptor site at exon 16 is predicted to activate a cryptic acceptor splice site 16 base pairs downstream of the mutated site. The alternate acceptor site would result in a frameshift and a termination codon one amino acid downstream of the frameshift [62–64]. The patient was born with polydactyly in the right foot, brachycephaly, moderate macrocephaly, and a unilateral supernumerary rib on the right side. He also presented abnormal facial features including a high, narrow palate, bulbous nose, protruding ears, and long eyelashes. He presented IGHD (7.0 ng/mL after stimulation test), but CPHD could not be discarded, since he also showed cryptorchidism. He suffered from global developmental delay, including dyslalia and tip-toe gait.

The identification of potential pathogenic variants in two candidate genes illustrates the utility of deep phenotyping of knockout mice to identify new candidate genes for congenital hypopituitarism.

## Discussion

Next-generation sequencing has revolutionized human genetics by enabling whole exome and whole genome sequencing of patients with congenital diseases, including CH, to identify variants associated with the disease. While candidate variants have become significantly easier to find, it is still difficult to determine the functional significance of VUS, especially in genes with no or limited association with the disease or tissue of interest. The international effort to knock out every gene in the mouse genome provides a rich source of mouse models to test the function of genetic lesions in organogenesis [65]. The DMDD effort to provide high-resolution images of embryonic lethal mouse embryos enabled a detailed assessment of gene function during organogenesis. Using the available data, we have identified a candidate gene list of 51 genes that are likely to function in pituitary gland organogenesis as pituitary malformations are observed in the embryonic lethal embryos. Among the genes analyzed by DMDD is *Tcf7l2*, which affects pituitary gland development by expanding Rathke’s pouch, and it has a pituitary malformation annotated by DMDD, which validates our screening approach. All the candidate genes are expressed in the tissues that can affect pituitary gland development, namely the ventral diencephalon, the anterior lobe of the pituitary gland, and the mesenchyme adjacent to the pituitary.

In addition to *Tcf7l2*, several genes, *Prop1*, *Wnt5a*, *Tle5*, *Sox3*, *Hesx1*, and others, have been identified that affect pituitary gland shape, including bifurcations of Rathke’s pouch and multiple invaginations of the pouch (reviewed in [66]). As with these known genes, the anterior lobe dysmorphologies observed in the candidate genes could be generated from multiple mechanisms. Continued proliferation in the stem cell niche could generate an overgrowth phenotype. A failure in an epithelial-to-mesenchymal-like transition could keep cells in the epithelial area and result in dysmorphology. The overgrowth phenotype can also be generated from excessive paracrine signaling from the ventral diencephalon that recruits too many pituitary progenitors initially or by stimulating excessive proliferation. A change in cell fate from an intermediate lobe identity to an anterior lobe identity could result in a fusion of the intermediate lobe with the anterior lobe across the cleft, as has been suggested when Notch signaling is stimulated in the intermediate lobe [67].

We used the candidate gene list and identified a likely pathogenic heterozygous variant in *MORC2* in a patient with CH with syndromic features. *MORC2* heterozygous variants occur in axonal Charcot–Marie–Tooth disease type 2Z, which is characterized by progressive peripheral neuropathy [68]. More recently heterozygous *MORC2* variants were found in individuals with DIGFAN syndrome (Developmental delay, impaired growth, dysmorphic facies, and axonal neuropathy), and endocrine abnormalities were observed in some patients [69]. The clinical phenotype of the patient reported here is consistent with other individuals with DIGFAN syndrome. It will be interesting to determine whether other genes associated with Charcot–Marie–Tooth disease are also associated with pituitary abnormalities in the future. For example, *POLR3B* has been associated with both Charcot–Marie–Tooth disease and syndromic hypogonadotropic hypogonadism [70, 71].

We also identified a pathogenic variant in *SETD5* in a patient with CH. SETD5 (or KIAA1757) contains a (Su(var)3–9, enhancer-of-zeste, trithorax) methyltransferase domain that catalyzes H3K36me3, and heterozygous loss of function mutations in humans are associated with intellectual disability and autism [63, 72]. Heterozygous loss of function in mice causes moderate growth insufficiency of indeterminate cause, evident by 2–3 weeks postnatally and persisting into adulthood. Brain weight to body weight ratios were elevated in mutants, indicative of macrocephaly, and their social and cognitive characteristics reflect those observed in patients. This suggests that the mouse is an excellent model for understanding the clinical features in patients. *Setd5* is highly expressed in Rathke's pouch and in neural tissue during development, and the homozygous mutant mice have an expanded, dysmorphic stem cell niche in Rathke's pouch and hypoplasia of the developing posterior lobe. These features are consistent with a potential role in suppressing stem cell proliferation and promoting transition to differentiation as observed in neuronal stem cell cultures. Haploinsufficiency for *Setd5* causes widespread alterations in neuronal gene expression, including delayed transcriptional elongation and impaired splicing. It will be intriguing to examine the effects of *Setd5* deficiency on pituitary stem cell differentiation and gene expression, providing insight into the mechanism of hypopituitarism in some patients.

The power of the knockout mouse project is that each knockout mouse line is cryopreserved and available to pursue a more in-depth functional assessment of *Morc2a*, *Setd5*, and other genes involved in pituitary gland organogenesis.

The expanded list of candidate genes identified novel molecular pathways that impact pituitary gland development. GO term clustering analysis identified molecular pathways, including ciliary function, amino acid metabolism, and epigenetics.

### Cilia proteins with potential roles in pituitary gland function.

**Fig. 5:**
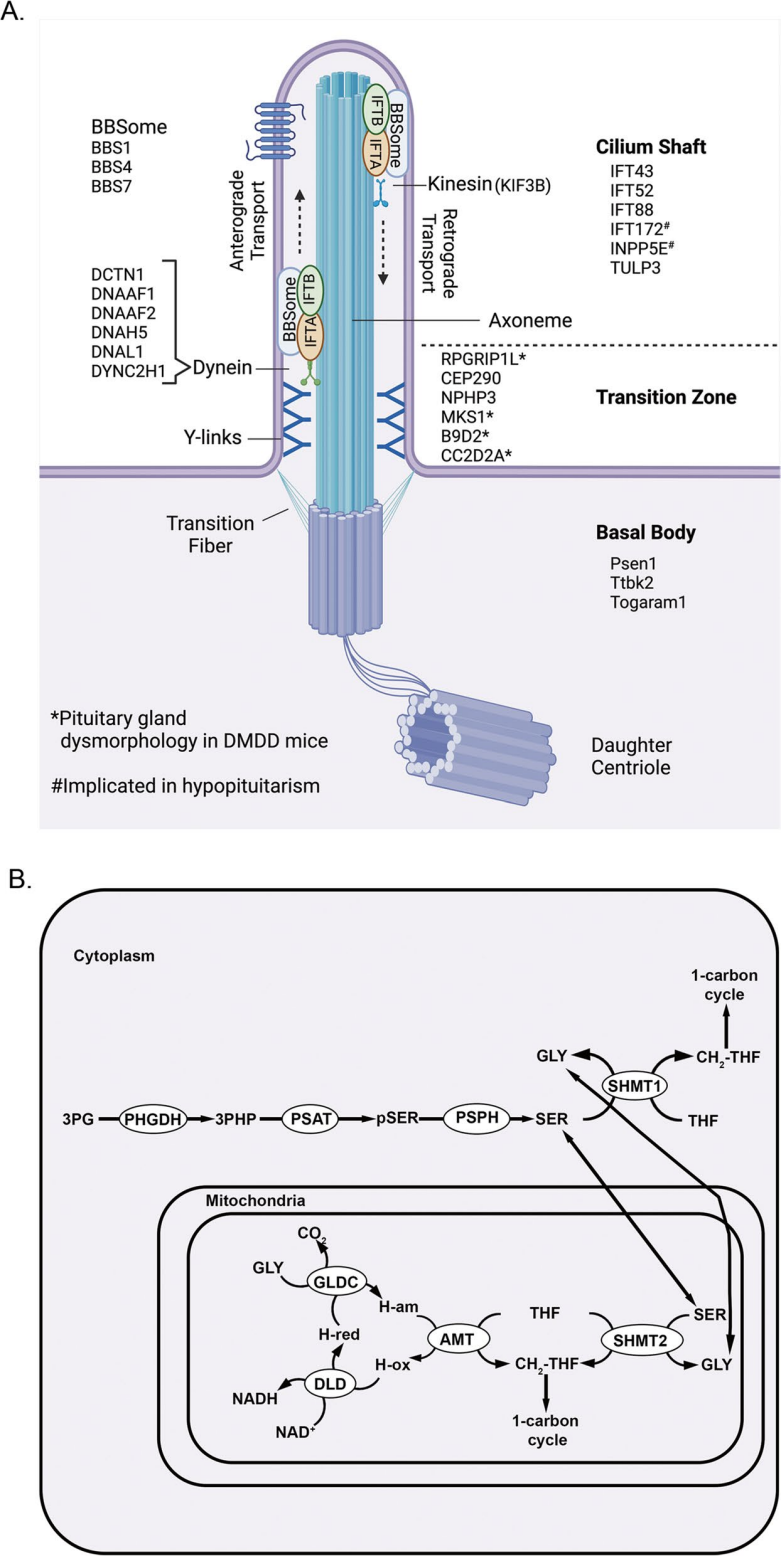
**A** Schematic of a primary cilium. Primary cilia consist of an axoneme extending from the apical surface of cells and a basal body. Transition fibers connect the basal body to the plasma membrane. Primary cilia form from centrosomes when the mother centriole becomes the basal body while retaining its attachment to the daughter centriole. The transition zone is located between the axoneme and the basal body and contains Y-shaped linkages that connect the microtubules to the plasma membrane. The transition zone is important for regulating transport of molecules into and out of the cilium. IFT-A and IFT-B are polymeric complexes that cooperate with the molecular motors, kinesin, and dynein, to transport cilia molecules. The role of the BBSome is not well understood, but studies suggest it has some role in cilial trafficking. *Analysis of HREM images from DMDD identifies pituitary hypoplasia in mice deficient in these genes. ^#^Patients have been identified who have mutations in these genes and exhibit ciliopathy phenotypes as well as hypopituitarism. This panel created using Biorender.com. (B) Schematic of glycine and serine metabolism. In the cytoplasm, the enzymes PHGDH, PSAT, and PSPH convert 3-phosphoglycerate into serine. SHMT1 can convert serine into glycine, while generating CH2-THF in the 1-carbon cycle. Products from the 1-carbon cycle are used for purine and methionine synthesis. In the mitochondria, SHMT2 can convert serine into glycine, while generating CH2-THF in the 1-carbon cycle. The glycine cleavage system, consisting of the enzymes GLDC, AMT, and DLD, also generates CH_2_-THF for the 1-carbon cycle. Abbreviations GLY (glycine), SER (serine), 3PG (3-phosphoglycerate), 3PHP (3-phosphohydroxypyruvate), pSER (phosphoserine), H-am (H-protein aminomethylated), H-ox (H-protein oxidized), H-red (H-protein reduced), THF (tetrahydrofolate), CH_2_-THF (5,10-methylene tetrafhydrofolate)

Primary cilia are cellular extensions that are thought to be immotile (Fig. 5A) [73]. They can sense changes in the extracellular milieu and communicate to the cell through various signaling pathways, including SHH and WNT. Because of these actions, primary cilia play important roles in development, and impairment of their function can result in a family of diseases referred to as ciliopathies. Ciliopathies consist of a range of developmental and degenerative conditions including retinal degeneration, anosmia, renal, hepatic, and pancreatic cyst formation, cleft palate, and polydactyly [73–75].

There is evidence supporting a role for ciliopathy genes in pituitary gland development and function [76, 77]. Within our candidate gene list are seven genes related to cilia function, *Mks1*, *B9d2*, *Rpgrip1l*, *Cc2d2a*, *Ehd1*, *Sh3pxd2a*, and *Ezr*. MKS1 is a component of the primary cilia and is mutated in Meckel syndrome type 1 and Bardet-Biedl syndrome type 13. *IFT172* variants have been associated with CH and Bardet-Biedl syndrome type 20 [73], and *TMEM67* variants have been associated with CH and Meckel/Joubert syndrome [74], and there is significant genetic overlap between these syndromes and Bardet-Biedl. In addition, whole exome sequencing of a patient with pituitary stalk interruption syndrome identified a pathogenic variant in *CC2D2A*, which is associated with absent corpus callosum [78], and a patient with Joubert syndrome, nephronophthisis, and pituitary agenesis has a splice acceptor site variant in *RPGRIP1L* [79]. It will be interesting to determine whether other genes associated with these syndromes will be found in CH patients with few syndromic features.

Cilia have been observed in the pituitary gland [80–83], and the cilia in stem cells lining the pituitary cleft have a 9 + 2 microtubule pattern, suggesting they are motile cilia [81]. Primary cilia also have been identified on somatotropes, lactotropes, and melanotropes [80, 81, 83]. Interestingly, receptors for dopamine and somatostatin are enriched in primary cilia in these cells, suggesting an important sensory role in hormone secretion [14, 15, 82, 83] Given the essential function of SHH signaling and ciliary function for midline specification, it is more likely that variants in ciliary proteins will present with CH in association with syndromes such as Joubert and Bardet-Biedl; however, isolated CH cannot be ruled out as a patient with a nonsense variant in *GLI2* presented with CH, but without the polydactyly usually observed in Culler-Jones syndrome [84]. Future studies will be imperative for uncovering the contribution of primary cilia to pituitary gland development and function and identifying the underlying mechanisms that lead to hypopituitarism.

### Serine and glycine metabolism and pituitary gland development

PHGDH, PSAT1, and PSPH function in the metabolic pathway that converts 3-phosphoglycerate into L-serine (Fig. 5B). Deficiencies in any of these enzymes result in low levels of serine and glycine in plasma and cerebrospinal fluid, which are associated with microcephaly, psychomotor retardation, and seizures (OMIM 601815, 610,992, and 614,023). GLDC is an enzyme, also known as the P protein, that operates in the mitochondrial glycine cleavage system. The glycine cleavage system produces 5,10-methylenetetrahydrofolate (CH_2_-THF), which is part of the one carbon (1C) cycle necessary for purine and methionine synthesis. Mutations in *GLDC* are the most common cause of glycine encephalopathy (GCE), or nonketotic hyperglycinemia, which is frequently postnatal lethal and characterized by lethargy, hypotonia, myoclonic jerks, and apnea [85]. The 1C cycle also receives carbon inputs from serine metabolism [86]. Serine can be converted to glycine and vice versa by the enzymes SHMT1 in the cytoplasm and SHMT2 in the mitochondria. During the conversion from serine to glycine, both enzymes will move carbon from the serine side chain to tetrahydrofolate (THF) to generate CH_2_-THF. Mutations in SHMT2 result in neurodevelopmental disorders with cardiomyopathy, spasticity, and brain abnormalities (NEDCASB) [87]. The brain abnormalities include microcephaly and corpus callosum hypoplasia. Pituitary defects have not been reported for any component of these metabolic pathways.

Functional analysis of *Psat1*^*−/−*^, *Psph*^*−/−*^, *Gldc*^*−/−*^ mouse models is necessary to determine how serine and glycine metabolism are linked to the observed pituitary malformations. The phenotype of the *Psat1*^*−/−*^, *Psph*^*−/−*^*,* and *Gldc*^*−/−*^ pituitaries is suggestive of increased paracrine signaling from the pituitary organizer in the ventral diencephalon to form Rathke’s pouch. There are several possibilities that could link these metabolic pathways and pituitary organizer signaling. The 1C cycle is important for S-adenosyl methionine (SAM) and methionine synthesis. SAM is a substrate used as a methyl donor for DNA and histone methylation. One possibility is that the metabolic inputs to epigenetic transcriptional regulation are disrupted in *Psat1*^*−/−*^, *Psph*^*−/−*^, and *Gldc*^*−/−*^ embryos and result in transcriptional changes that expanded the expression of the paracrine signals generated in the ventral diencephalon. In addition to SAM and methionine, the 1C cycle is necessary for purine synthesis. Careful analysis of *Gldc*^*−/−*^ embryos, which have neural tube defects, suggests that purine synthesis, not SAM or methionine production, is necessary for neural tube closure [88]. Purine synthesis is necessary for cellular proliferation, which is critical for embryogenesis. However, a connection between ventral diencephalon proliferation and paracrine signaling is unknown. Another possibility is that purine synthesis from the 1C cycle is necessary for purinergic signaling in the ventral diencephalon to regulate the signaling factors generated by the pituitary organizer. Purinergic receptors are expressed throughout the hypothalamus and pituitary gland and purinergic signaling is known to regulate the release of both hypothalamic-releasing hormones and pituitary hormones [89]. A role for purinergic signaling in ventral diencephalon pattern formation has not been determined. However, purinergic signaling can stimulate BMP4 expression in osteoblasts [90], FGF2 expression in the olfactory epithelium [91], and inhibits SHH in the chick neural tube [92], suggesting that if active in the developing ventral diencephalon purinergic signaling may regulate the signaling molecules necessary for pituitary progenitor induction.

### Epigenetics

Both the known CH-causing genes and the candidate CH genes are enriched for genes involved in transcriptional regulation. The genes associated with CH include transcription factors, including *Sox2*, *Prop1*, and *Pou1f1*, whose characterization in mouse pituitary gland development has generated our current understanding of the transcriptional hierarchy necessary for pituitary cell specification [93]. In comparison, the candidate gene list does not contain many transcription factors but instead is enriched in genes whose protein products are involved in chromatin binding and chromatin modification. We consider these genes epigenetic regulators, which provide novel mechanisms for understanding the transcriptional regulation necessary for pituitary gland morphogenesis and cell specification. Among these epigenetic regulators are *Jarid2*, *Kmt5b*, *Arid2*, and *Kat14*, which have varied functions in transcriptional regulation. JARID2 is a Polycomb Repressive Complex 2 (PRC2) cofactor that recognizes ubiquitinated H2A lysine 119 (H2AK119u1), resulting in PRC2 recruitment to the chromatin and transcriptional silencing [94]. Autosomal dominant mutations in *JARID2* are known to cause developmental delay with variable intellectual disability and dysmorphic facies in humans [95], and homozygous null mouse mutants are embryonic lethal with neural tube defects [96]. KMT5B is a histone methyltransferase that methylates monomethylated lysine 20 on histone H4 to generate a dimethyl, which is then trimethylated, resulting in highly condensed heterochromatin [97]. Autosomal dominant mutations in humans result in intellectual developmental disorder [98, 99], while homozygous null mice are perinatal lethal with alveolar defects [100]. In contrast to the transcriptional repressive effects of JARID2 and KMT5B, ARID2 opens chromatin as part of the PBAF chromatin-remodeling complex, leading to transcriptional activation [100]. Coffin-Siris syndrome 6 is caused by heterozygous mutations in ARID2 [101, 102]. *Arid2*^*−/−*^ mice are embryonic lethal between e12.5 and e14.5 with congenital heart defects [103]. KAT14 is necessary for histone acetylation, which is also associated with transcriptional activation [104]. *Kat14*^*−/−*^ mouse embryos are embryonic lethal by e11.5 [104], suggesting that the knockout first allele is hypomorphic as the DMDD phenotyping was performed on e14.5 embryos. Hypomorphic alleles may be beneficial for identifying additional organs, such as the pituitary gland, where transcriptional regulation by chromatin modifiers is essential for organogenesis.

As discussed above, autosomal dominant mutations in *MORC2* cause DIGFAN syndrome and Charcot–Marie–Tooth disease [69]. MORC2 functions with the human silencing hub complex (HUSH) [105], which results in trimethylation of histone 3 on lysine 9 [106]. The HUSH complex is known to silence repetitive elements, such as L1 retrotransposons, but also functions to silence the repetitive-like protocadherin gene cluster [107]. Loss of *Morc2a* in the mouse nervous system results in protocadherin expression and increased brain size and brain dysmorphology. Further analysis in the mouse pituitary gland will determine how HUSH silencing mediates transcriptional regulation during pituitary cell specification, potentially resulting in the endocrine disruptions observed in DIGFAN syndrome.

## Conclusions

The international effort to generate knockout mice and phenotyping data provides a wealth of information for the research community, which we have screened to identify 51 genes not previously associated with pituitary gland organogenesis. Almost 25% of the embryonic lethal and sub-viable mice screened in this analysis have a pituitary malformation. With more than 1400 lethal and sub-viable strains already identified, the probability of identifying additional genes with roles in pituitary gland organogenesis is high. The candidate CH genes identified here provide entry points to characterize new molecular pathways involved in pituitary gland development and additional candidate genes to screen for variants associated with human CH, SOD, HPE, and other syndromic disorders. The identification of variants in *MORC2* and *SETD5* in patients with CH demonstrates the usefulness of this candidate gene list.

Mouse models have been excellent predictors of human disease; however, there will be differences, especially given the large number of candidate genes and the limited characterization that has occurred. Mouse pituitary gland malformations do not guarantee that mice or humans with variations in the same genes will have a pituitary hormone deficiency. For instance, *Wnt5a*^*−/−*^ mice have a dysmorphic pituitary gland but do not have an obvious hormone deficiency by late gestation [108]. The severity of the pituitary disease may also differ as the mouse alleles are designed to be null alleles, while some human variants are likely to be missense, potentially generating a hypomorphic allele instead of a null allele. Many of the candidate genes have a variable penetrance, which is surprising given that the mice are inbred, which reduces the possibility of phenotypic variability resulting from genetic modifiers. Phenotypic variability, including variable penetrance, is commonly observed in human midline disorders and is characteristic of multifactorial syndromes like HPE, SOD, and CH [109–111]. The phenotypic variability observed in both mice and humans highlights the role that chance, stochastic factors, genetic modifiers, and environmental exposures have in determining phenotype [112]. Many of the candidate genes identified here are associated with severe malformations in other tissues. Human fetuses with similar homozygous null mutations are not likely to survive and will not be characterized with a pituitary deficiency. However, less severe, hypomorphic alleles may present with pituitary deficiency as part of a complex syndrome. With 32 of the candidate genes associated with human disease, we encourage clinicians who care for patients with these diseases to consider possible endocrine deficiencies in their patients.

### Supplementary Information

Additional file 1: Tabel S1. GO terms, KEGG pathway, and Uniprot key words associated with candidate genes and known CH genes.

Additional file 2: Figure S1. scRNA-seq quality control data.

Additional file 3: Table S2. Annotated malformations observed in embryonic lethal embryos of CH candidate genes.

Additional file 4: Table S3. DAVID Functional Annotation Clustering for the known CH genes and candidate genes.

Additional file 5: Figure S2. Pituitary gland malformations observed in candidate CH genes.

Additional file 6: Figure S3. Pituitary gland malformations observed in candidate CH genes.

Additional file 7: Figure S4. Candidate gene expression in the embryonic pituitary gland.

Additional file 8: Figure S5. RNA in situ hybridization images captured from the GenePaint database.

Additional file 9: Figure S6. RNA in situ hybridization images captured from the GenePaint database.

## Data Availability

Requests for DMDD HREM data can be made to Dr. Stefan Geyer at the Medical University of Vienna, stefan.geyer@meduniwien.ac.at. Please allow three weeks for responses to reasonable requests. GenePaint is a database of gene expression patterns that is searchable by gene name, accession number, sequence homology, and site of expression, https://gp3.mpg.de/ [20]. Single-cell RNA sequencing data generated in association with this manuscript are deposited at GEO Datasets, with the series accession number GSE246211 (https://www.ncbi.nlm.nih.gov/geo/query/acc.cgi?acc=GSE246211) [30], and samples GSM7864906 (https://www.ncbi.nlm.nih.gov/geo/query/acc.cgi?acc=GSM7864906) [31] and GSM7864907 (https://www.ncbi.nlm.nih.gov/geo/query/acc.cgi?acc=GSM7864907) [32].

## Abbreviations

CH_2_-THF: 5,10-Methylenetetrahydrofolate
ACTH: Adrenocorticotropic hormone
ACMG: American College of Medical Genetics and Genomics
CPHD: Combined pituitary hormone deficiency
CH: Congenital hypopituitarism
DAVID: Database for Annotation, Visualization, and Integrated Discovery
DMDD: Deciphering Mechanisms of Developmental Disorders
DIGFAN: Developmental delay, impaired growth, dysmorphic facies, and axonal neuropathy
e: Embryonic day of development
FSH: Follicle-stimulating hormone
GO: Gene ontology
GCE: Glycine encephalopathy
GH: Growth hormone
GHD: Growth hormone deficiency
HREM: High-resolution episcopic microscopy
HPE: Holoprosencephaly
HH: Hypogonadotropic hypogonadism
IP3: Inositol 1,4,5 trisphosphate
IMKC: International Mouse Knockout Consortium
IMPC: International Mouse Phenotyping Consortium
IGHD: Isolated growth hormone deficiency
KOMP: Knockout mouse project
KEGG: Kyoto Encyclopedia of Genes and Genomes
LH: Luteinizing hormone
MSH: Melanocyte-stimulating hormone
NEDCASB: Neurodevelopmental disorder with cardiomyopathy, spasticity, and brain abnormalities
1C: One carbon
OMIM: Online Mendelian Inheritance in Man
PRL: Prolactin
POMC: Proopiomelanocortin
rhGH: Recombinant human growth hormone
RCF: Relative centrifugal force
SOD: Septo-optic dysplasia
THF: Tetrahydrofolate
TSH: Thyroid-stimulating hormone
VUS: Variants of uncertain significance
WES: Whole exome sequencing

## References

[1] Vishnopolska SA, Mercogliano MF, Camilletti MA, Mortensen AH, Braslavsky D, Keselman A (2021). Comprehensive identification of pathogenic gene variants in patients with neuroendocrine disorders. J Clin Endocrinol Metab.

[2] Fang Q, George AS, Brinkmeier ML, Mortensen AH, Gergics P, Cheung LY (2016). Genetics of combined pituitary hormone deficiency: roadmap into the genome era. Endocr Rev.

[3] Dusatkova P, Pfäffle R, Brown MR, Akulevich N, Arnhold IJ, Kalina MA (2016). Genesis of two most prevalent PROP1 gene variants causing combined pituitary hormone deficiency in 21 populations. Eur J Human Genet: EJHG.

[4] Gregory LC, Cionna C, Cerbone M, Dattani MT (2023). Identification of genetic variants and phenotypic characterization of a large cohort of patients with congenital hypopituitarism and related disorders. Genet Med.

[5] Blum WF, Klammt J, Amselem S, Pfäffle HM, Legendre M, Sobrier ML (2018). Screening a large pediatric cohort with GH deficiency for mutations in genes regulating pituitary development and GH secretion: Frequencies, phenotypes and growth outcomes. EBioMedicine.

[6] Bando H, Urai S, Kanie K, Sasaki Y, Yamamoto M, Fukuoka H (2022). Novel genes and variants associated with congenital pituitary hormone deficiency in the era of next-generation sequencing. Front Endocrinol (Lausanne).

[7] Richards S, Aziz N, Bale S, Bick D, Das S, Gastier-Foster J (2015). Standards and guidelines for the interpretation of sequence variants: a joint consensus recommendation of the American College of Medical Genetics and Genomics and the Association for Molecular Pathology. Genet Med.

[8] De Rienzo F, Mellone S, Bellone S, Babu D, Fusco I, Prodam F (2015). Frequency of genetic defects in combined pituitary hormone deficiency: a systematic review and analysis of a multicentre Italian cohort. Clin Endocrinol (Oxf).

[9] Jullien N, Saveanu A, Vergier J, Marquant E, Quentien MH, Castinetti F (2021). Clinical lessons learned in constitutional hypopituitarism from two decades of experience in a large international cohort. Clin Endocrinol (Oxf).

[10] Gregory LC, Dattani MT. The Molecular Basis of Congenital Hypopituitarism and Related Disorders. J Clin Endocrinol Metab. 2020;105(6). 10.1210/clinem/dgz184 31702014

[11] Rizzoti K, Lovell-Badge R (2005). Early development of the pituitary gland: induction and shaping of Rathke's pouch. Rev Endocr Metab Disord.

[12] Carreno G, Apps JR, Lodge EJ, Panousopoulos L, Haston S, Gonzalez-Meljem JM (2017). Hypothalamic sonic hedgehog is required for cell specification and proliferation of LHX3/LHX4 pituitary embryonic precursors. Development.

[13] Fauquier T, Rizzoti K, Dattani M, Lovell-Badge R, Robinson IC (2008). SOX2-expressing progenitor cells generate all of the major cell types in the adult mouse pituitary gland. Proc Natl Acad Sci U S A.

[14] Schwind JL (1928). The development of the hypophysis cerebri of the albino rat. Am J Anatom.

[15] Garcia-Lavandeira M, Quereda V, Flores I, Saez C, Diaz-Rodriguez E, Japon MA (2009). A GRFa2/Prop1/stem (GPS) cell niche in the pituitary. PLoS ONE.

[16] Cheung LY, Davis SW, Brinkmeier ML, Camper SA, Perez-Millan MI. Regulation of pituitary stem cells by epithelial to mesenchymal transition events and signaling pathways. Mol Cell Endocrinol. 2016. 10.1016/j.mce.2016.09.016 PMC5590650 27650955

[17] Mohun T, Adams DJ, Baldock R, Bhattacharya S, Copp AJ, Hemberger M (2013). Deciphering the Mechanisms of Developmental Disorders (DMDD): a new programme for phenotyping embryonic lethal mice. Dis Model Mech.

[18] Wilson R, McGuire C, Mohun T (2016). Deciphering the mechanisms of developmental disorders: phenotype analysis of embryos from mutant mouse lines. Nucleic Acids Res.

[19] Brinkmeier ML, Potok MA, Davis SW, Camper SA (2007). TCF4 deficiency expands ventral diencephalon signaling and increases induction of pituitary progenitors. Dev Biol.

[20] Visel A, Thaller C, Eichele G (2004). GenePaint.org: an atlas of gene expression patterns in the mouse embryo. Nucleic Acids Res..

[21] Diez-Roux G, Banfi S, Sultan M, Geffers L, Anand S, Rozado D (2011). A high-resolution anatomical atlas of the transcriptome in the mouse embryo. PLoS Biol.

[22] Eichele G, Diez-Roux G (2011). High-throughput analysis of gene expression on tissue sections by in situ hybridization. Methods.

[23] da Huang W, Sherman BT, Lempicki RA (2009). Systematic and integrative analysis of large gene lists using DAVID bioinformatics resources. Nat Protoc.

[24] Sherman BT, Hao M, Qiu J, Jiao X, Baseler MW, Lane HC (2022). DAVID: a web server for functional enrichment analysis and functional annotation of gene lists (2021 update). Nucleic Acids Res.

[25] Ashburner M, Ball CA, Blake JA, Botstein D, Butler H, Cherry JM (2000). Gene ontology: tool for the unification of biology. The Gene Ontology Consortium. Nat Genet.

[26] Aleksander SA, Balhoff J, Carbon S, Cherry JM, Drabkin HJ, Ebert D, et al. The Gene Ontology knowledgebase in 2023. Genetics. 2023;224(1). 10.1093/genetics/iyad031 PMC10158837 36866529

[27] UniProt: the Universal Protein Knowledgebase in 2023. Nucleic Acids Res. 2023;51(D1):D523-d31. 10.1093/nar/gkac1052 PMC9825514 36408920

[28] Kanehisa M, Furumichi M, Sato Y, Kawashima M, Ishiguro-Watanabe M (2023). KEGG for taxonomy-based analysis of pathways and genomes. Nucleic Acids Res.

[29] Hogan B, Beddington R, Costantini F, Lacey E (1994). Manipulating the mouse embryo: a laboratory manual.

[30] Martinez-Mayer J BM, O’Connell SP, Ukagwu A, Marti MA, Miras M, Forclaz MV, Benzrihen MG, Cheung LY, Camper SA, Ellsworth BS, Raetzman LT, Pérez Millán MI, Davis SW. Knockout mice with pituitary malformations help identify human cases of hypopituitarism. GSE246211, NCBI Gene Expression Ominbus. 2023. https://www.ncbi.nlm.nih.gov/geo/query/acc.cgi?acc=GSE246211 10.1186/s13073-024-01347-y PMC11140907 38822427

[31] Martinez-Mayer J BM OCS, Ukagwu A, Marti MA, Miras M, Forclaz MV, Benzrihen MG, Cheung LY, Camper SA, Ellsworth BS, Raetzman LT, Pérez Millán MI, Davis SW. e12.5 pituitary gland from a retinoic acid reporter mouse (RARE-LacZ JAX strain #008477). GSM786906, NCBI Gene Expression Omnibus. 2023. https://www.ncbi.nlm.nih.gov/geo/query/acc.cgi?acc=GSM7864906

[32] Martinez-Mayer J BM OCS, Ukagwu A, Marti MA, Miras M, Forclaz MV, Benzrihen MG, Cheung LY, Camper SA, Ellsworth BS, Raetzman LT, Pérez Millán MI, Davis SW. Two pooled e14.5 pituitary glands from C57BL6 strain. GMS7864907, NCBI Gene Expression Omnibus. 2023. https://www.ncbi.nlm.nih.gov/geo/query/acc.cgi?acc=GSM7864907

[33] Gergics P, Smith C, Bando H, Jorge AAL, Rockstroh-Lippold D, Vishnopolska SA (2021). High-throughput splicing assays identify missense and silent splice-disruptive POU1F1 variants underlying pituitary hormone deficiency. Am J Hum Genet.

[34] Martinez-Mayer J, Vishnopolska S, Perticarari C, Garcia LI, Hackbartt M, Martinez M, et al. Exome Sequencing has a high diagnostic rate in sporadic congenital hypopituitarism and reveals novel candidate genes. J Clin Endocrinol Metab. 2024. 10.1210/clinem/dgae320 38717911

[35] Li H, Durbin R (2009). Fast and accurate short read alignment with Burrows-Wheeler transform. Bioinformatics.

[36] McKenna A, Hanna M, Banks E, Sivachenko A, Cibulskis K, Kernytsky A (2010). The genome analysis toolkit: a MapReduce framework for analyzing next-generation DNA sequencing data. Genome Res.

[37] Van der Auwera GA, Carneiro MO, Hartl C, Poplin R, Del Angel G, Levy-Moonshine A (2013). From FastQ data to high confidence variant calls: the Genome Analysis Toolkit best practices pipeline. Curr Protoc Bioinform..

[38] Wang K, Li M, Hakonarson H (2010). ANNOVAR: functional annotation of genetic variants from high-throughput sequencing data. Nucleic Acids Res.

[39] Sherry ST, Ward MH, Kholodov M, Baker J, Phan L, Smigielski EM (2001). dbSNP: the NCBI database of genetic variation. Nucleic Acids Res.

[40] Karczewski KJ, Francioli LC, Tiao G, Cummings BB, Alföldi J, Wang Q (2020). The mutational constraint spectrum quantified from variation in 141,456 humans. Nature.

[41] Adzhubei I, Jordan DM, Sunyaev SR. Predicting functional effect of human missense mutations using PolyPhen-2. Current protocols in human genetics. 2013;Chapter 7:Unit7.20. 10.1002/0471142905.hg0720s76 PMC4480630 23315928

[42] Ng PC, Henikoff S (2003). SIFT: Predicting amino acid changes that affect protein function. Nucleic Acids Res.

[43] Schwarz JM, Cooper DN, Schuelke M, Seelow D (2014). MutationTaster2: mutation prediction for the deep-sequencing age. Nat Methods.

[44] Jaganathan K, Kyriazopoulou Panagiotopoulou S, McRae JF, Darbandi SF, Knowles D, Li YI (2019). Predicting splicing from primary sequence with deep learning. Cell.

[45] Ye J, Coulouris G, Zaretskaya I, Cutcutache I, Rozen S, Madden TL (2012). Primer-BLAST: a tool to design target-specific primers for polymerase chain reaction. BMC Bioinformatics.

[46] Ayadi A, Birling MC, Bottomley J, Bussell J, Fuchs H, Fray M (2012). Mouse large-scale phenotyping initiatives: overview of the European Mouse Disease Clinic (EUMODIC) and of the Wellcome Trust Sanger Institute Mouse Genetics Project. Mamm Genome.

[47] Waterhouse AM, Procter JB, Martin DM, Clamp M, Barton GJ (2009). Jalview Version 2–a multiple sequence alignment editor and analysis workbench. Bioinformatics.

[48] Douse CH, Bloor S, Liu Y, Shamin M, Tchasovnikarova IA, Timms RT (2018). Neuropathic MORC2 mutations perturb GHKL ATPase dimerization dynamics and epigenetic silencing by multiple structural mechanisms. Nat Commun.

[49] Webb B, Sali A (2016). Comparative protein structure modeling using MODELLER. Curr Protoc Bioinform..

[50] Humphrey W, Dalke A, Schulten K (1996). VMD: visual molecular dynamics. J Mol Graph..

[51] Brinkmeier ML, Potok MA, Cha KB, Gridley T, Stifani S, Meeldijk J (2003). TCF and Groucho-related genes influence pituitary growth and development. Mol Endocrinol.

[52] Wilson R, Geyer SH, Reissig L, Rose J, Szumska D, Hardman E (2016). Highly variable penetrance of abnormal phenotypes in embryonic lethal knockout mice. Wellcome Open Res.

[53] Ho EK, Stearns T. Hedgehog signaling and the primary cilium: implications for spatial and temporal constraints on signaling. Development. 2021;148(9). 10.1242/dev.195552 PMC8126410 33914866

[54] Locasale JW (2013). Serine, glycine and one-carbon units: cancer metabolism in full circle. Nat Rev Cancer.

[55] Roberts C, Sutherland HF, Farmer H, Kimber W, Halford S, Carey A (2002). Targeted mutagenesis of the Hira gene results in gastrulation defects and patterning abnormalities of mesoendodermal derivatives prior to early embryonic lethality. Mol Cell Biol.

[56] McClatchey AI (2014). ERM proteins at a glance. J Cell Sci.

[57] Albulym OM, Kennerson ML, Harms MB, Drew AP, Siddell AH, Auer-Grumbach M (2016). MORC2 mutations cause axonal Charcot-Marie-Tooth disease with pyramidal signs. Ann Neurol.

[58] Zhang N, Chen Y, Lu H, Zhao F, Alvarez RV, Goncearenco A (2020). MutaBind2: Predicting the impacts of single and multiple mutations on protein-protein interactions. iScience..

[59] Deciphering Developmental Disorders S (2017). Prevalence and architecture of de novo mutations in developmental disorders. Nature..

[60] Klee EW, Cousin MA, Pinto EVF, Morales-Rosado JA, Macke EL, Jenkinson WG (2021). Impact of integrated translational research on clinical exome sequencing. Genet Med.

[61] Klee EW, Cousin MA, Pinto EVF, Morales-Rosado JA, Macke EL, Jenkinson WG (2023). Impact of integrated translational research on clinical exome sequencing. Genet Med.

[62] Kuechler A, Zink AM, Wieland T, Lüdecke HJ, Cremer K, Salviati L (2015). Loss-of-function variants of SETD5 cause intellectual disability and the core phenotype of microdeletion 3p25.3 syndrome. Eur J Human Genet: EJHG..

[63] Grozeva D, Carss K, Spasic-Boskovic O, Parker MJ, Archer H, Firth HV (2014). De novo loss-of-function mutations in SETD5, encoding a methyltransferase in a 3p25 microdeletion syndrome critical region, cause intellectual disability. Am J Hum Genet.

[64] Powis Z, Farwell Hagman KD, Mroske C, McWalter K, Cohen JS, Colombo R (2018). Expansion and further delineation of the SETD5 phenotype leading to global developmental delay, variable dysmorphic features, and reduced penetrance. Clin Genet.

[65] Collins FS, Rossant J, Wurst W (2007). A mouse for all reasons. Cell.

[66] Rizzoti K (2010). Adult pituitary progenitors/stem cells: from in vitro characterization to in vivo function. Eur J Neurosci.

[67] Cheung L, Le Tissier P, Goldsmith SG, Treier M, Lovell-Badge R, Rizzoti K. NOTCH activity differentially affects alternative cell fate acquisition and maintenance. eLife. 2018;7. 10.7554/eLife.33318 PMC5889214 29578405

[68] Guillen Sacoto MJ, Tchasovnikarova IA, Torti E, Forster C, Andrew EH, Anselm I (2020). De novo variants in the ATPase module of MORC2 cause a neurodevelopmental disorder with growth retardation and variable craniofacial dysmorphism. Am J Hum Genet.

[69] Jacquier A, Roubille S, Lomonte P, Schaeffer L (2022). Microrchidia CW-Type Zinc Finger 2, a chromatin modifier in a spectrum of peripheral neuropathies. Front Cell Neurosci.

[70] Tétreault M, Choquet K, Orcesi S, Tonduti D, Balottin U, Teichmann M (2011). Recessive mutations in POLR3B, encoding the second largest subunit of Pol III, cause a rare hypomyelinating leukodystrophy. Am J Hum Genet.

[71] Bernard G, Vanderver A. POLR3-Related Leukodystrophy. In: Adam MP, Mirzaa GM, Pagon RA, Wallace SE, Bean LJH, Gripp KW, et al., editors. GeneReviews(®). Seattle (WA): University of Washington, Seattle

[72] Copyright © 1993–2023, University of Washington, Seattle. GeneReviews is a registered trademark of the University of Washington, Seattle. All rights reserved.; 1993.

[73] Sessa A, Fagnocchi L, Mastrototaro G, Massimino L, Zaghi M, Indrigo M (2019). SETD5 regulates chromatin methylation state and preserves global transcriptional fidelity during brain development and neuronal wiring. Neuron.

[74] Baker K, Beales PL (2009). Making sense of cilia in disease: the human ciliopathies. Am J Med Genet C Semin Med Genet..

[75] Wheway G, Nazlamova L, Hancock JT (2018). Signaling through the primary cilium. Front Cell Dev Biol.

[76] Andreu-Cervera A, Catala M, Schneider-Maunoury S (2021). Cilia, ciliopathies and hedgehog-related forebrain developmental disorders. Neurobiol Dis.

[77] Voutetakis A (2021). Pituitary stalk interruption syndrome. Handb Clin Neurol.

[78] Lodge EJ, Barrell WB, Liu KJ, Andoniadou CL (2024). The Fuzzy planar cell polarity protein (FUZ), necessary for primary cilium formation, is essential for pituitary development. J Anat.

[79] Zwaveling-Soonawala N, Alders M, Jongejan A, Kovacic L, Duijkers FA, Maas SM (2018). Clues for polygenic inheritance of pituitary stalk interruption syndrome from exome sequencing in 20 patients. J Clin Endocrinol Metab.

[80] Wolf MT, Saunier S, O'Toole JF, Wanner N, Groshong T, Attanasio M (2007). Mutational analysis of the RPGRIP1L gene in patients with Joubert syndrome and nephronophthisis. Kidney Int.

[81] Barnes BG (1961). Ciliated secretory cells in the pars distalis of the mouse hypophysis. J Ultrastruct Res.

[82] Correr S, Motta PM (1981). The rat pituitary cleft: a correlated study by scanning and transmission electron microscopy. Cell Tissue Res.

[83] Iwanaga T, Hozumi Y, Takahashi-Iwanaga H (2011). Immunohistochemical demonstration of dopamine receptor D2R in the primary cilia of the mouse pituitary gland. Biomed Res.

[84] Iwanaga T, Miki T, Takahashi-Iwanaga H (2011). Restricted expression of somatostatin receptor 3 to primary cilia in the pancreatic islets and adenohypophysis of mice. Biomed Res.

[85] França MM, Jorge AA, Carvalho LR, Costalonga EF, Vasques GA, Leite CC (2010). Novel heterozygous nonsense GLI2 mutations in patients with hypopituitarism and ectopic posterior pituitary lobe without holoprosencephaly. J Clin Endocrinol Metab.

[86] Kure S, Kato K, Dinopoulos A, Gail C, DeGrauw TJ, Christodoulou J (2006). Comprehensive mutation analysis of GLDC, AMT, and GCSH in nonketotic hyperglycinemia. Hum Mutat.

[87] Ducker GS, Rabinowitz JD (2017). One-Carbon Metabolism in Health and Disease. Cell Metab.

[88] Groza T, Gomez FL, Mashhadi HH, Muñoz-Fuentes V, Gunes O, Wilson R, et al. The International Mouse Phenotyping Consortium: comprehensive knockout phenotyping underpinning the study of human disease. Nucleic Acids Res. 2022. 10.1093/nar/gkac972 PMC9825559 36305825

[89] Leung KY, Pai YJ, Chen Q, Santos C, Calvani E, Sudiwala S (2017). Partitioning of one-carbon units in folate and methionine metabolism is essential for neural tube closure. Cell Rep.

[90] Bjelobaba I, Janjic MM, Stojilkovic SS (2015). Purinergic signaling pathways in endocrine system. Auton Neurosci.

[91] Ayala-Peña VB, Scolaro LA, Santillán GE (2013). ATP and UTP stimulate bone morphogenetic protein-2,-4 and -5 gene expression and mineralization by rat primary osteoblasts involving PI3K/AKT pathway. Exp Cell Res.

[92] Jia C, Cussen AR, Hegg CC (2011). ATP differentially upregulates fibroblast growth factor 2 and transforming growth factor α in neonatal and adult mice: effect on neuroproliferation. Neuroscience.

[93] Yatsuzuka A, Hori A, Kadoya M, Matsuo-Takasaki M, Kondo T, Sasai N. GPR17 is an essential regulator for the temporal adaptation of sonic hedgehog signalling in neural tube development. Development. 2019;146(17). 10.1242/dev.176784 31444216

[94] Davis SW, Ellsworth BS, Perez Millan MI, Gergics P, Schade V, Foyouzi N (2013). Pituitary gland development and disease: from stem cell to hormone production. Curr Top Dev Biol.

[95] Cooper S, Grijzenhout A, Underwood E, Ancelin K, Zhang T, Nesterova TB (2016). Jarid2 binds mono-ubiquitylated H2A lysine 119 to mediate crosstalk between Polycomb complexes PRC1 and PRC2. Nat Commun.

[96] Verberne EA, Goh S, England J, van Ginkel M, Rafael-Croes L, Maas S (2021). JARID2 haploinsufficiency is associated with a clinically distinct neurodevelopmental syndrome. Genet Med.

[97] Takeuchi T, Yamazaki Y, Katoh-Fukui Y, Tsuchiya R, Kondo S, Motoyama J (1995). Gene trap capture of a novel mouse gene, jumonji, required for neural tube formation. Genes Dev.

[98] Southall SM, Cronin NB, Wilson JR (2014). A novel route to product specificity in the Suv4-20 family of histone H4K20 methyltransferases. Nucleic Acids Res.

[99] Iossifov I, O'Roak BJ, Sanders SJ, Ronemus M, Krumm N, Levy D (2014). The contribution of de novo coding mutations to autism spectrum disorder. Nature.

[100] Schotta G, Sengupta R, Kubicek S, Malin S, Kauer M, Callén E (2008). A chromatin-wide transition to H4K20 monomethylation impairs genome integrity and programmed DNA rearrangements in the mouse. Genes Dev.

[101] Yan Z, Cui K, Murray DM, Ling C, Xue Y, Gerstein A (2005). PBAF chromatin-remodeling complex requires a novel specificity subunit, BAF200, to regulate expression of selective interferon-responsive genes. Genes Dev.

[102] Shang L, Cho MT, Retterer K, Folk L, Humberson J, Rohena L (2015). Mutations in ARID2 are associated with intellectual disabilities. Neurogenetics.

[103] Bramswig NC, Caluseriu O, Lüdecke HJ, Bolduc FV, Noel NC, Wieland T (2017). Heterozygosity for ARID2 loss-of-function mutations in individuals with a Coffin-Siris syndrome-like phenotype. Hum Genet.

[104] He L, Tian X, Zhang H, Hu T, Huang X, Zhang L (2014). BAF200 is required for heart morphogenesis and coronary artery development. PLoS ONE.

[105] Guelman S, Kozuka K, Mao Y, Pham V, Solloway MJ, Wang J (2009). The double-histone-acetyltransferase complex ATAC is essential for mammalian development. Mol Cell Biol.

[106] Tchasovnikarova IA, Timms RT, Douse CH, Roberts RC, Dougan G, Kingston RE (2017). Hyperactivation of HUSH complex function by Charcot-Marie-Tooth disease mutation in MORC2. Nat Genet.

[107] Tchasovnikarova IA, Timms RT, Matheson NJ, Wals K, Antrobus R, Göttgens B (2015). GENE SILENCING. Epigenetic silencing by the HUSH complex mediates position-effect variegation in human cells. Science..

[108] Hagelkruys A, Horrer M, Taubenschmid-Stowers J, Kavirayani A, Novatchkova M, Orthofer M (2022). The HUSH complex controls brain architecture and protocadherin fidelity. Sci Adv..

[109] Cha KB, Douglas KR, Potok MA, Liang H, Jones SN, Camper SA (2004). WNT5A signaling affects pituitary gland shape. Mech Dev.

[110] Petryk A, Graf D, Marcucio R (2015). Holoprosencephaly: signaling interactions between the brain and the face, the environment and the genes, and the phenotypic variability in animal models and humans. Wiley Interdiscip Rev Dev Biol.

[111] Bosch IAL, Katugampola H, Dattani MT (2020). Congenital hypopituitarism during the neonatal period: epidemiology, pathogenesis, therapeutic options, and outcome. Front Pediatr.

[112] Reis LM, Seese S, Maheshwari M, Basel D, Weik L, McCarrier J, et al. Novel genetic diagnoses in septo-optic dysplasia. Genes (Basel). 2022;13(7). 10.3390/genes13071165 PMC9320703 35885948

[113] Czyz W, Morahan JM, Ebers GC, Ramagopalan SV (2012). Genetic, environmental and stochastic factors in monozygotic twin discordance with a focus on epigenetic differences. BMC Med.

