# Supplementary figures and images for: Knockout mice with pituitary malformations help identify human cases of hypopituitarism
Source: Genome Med. 2024 May 31;16:75. doi: 10.1186/s13073-024-01347-y (PMC11140907; doi:10.1186/s13073-024-01347-y)

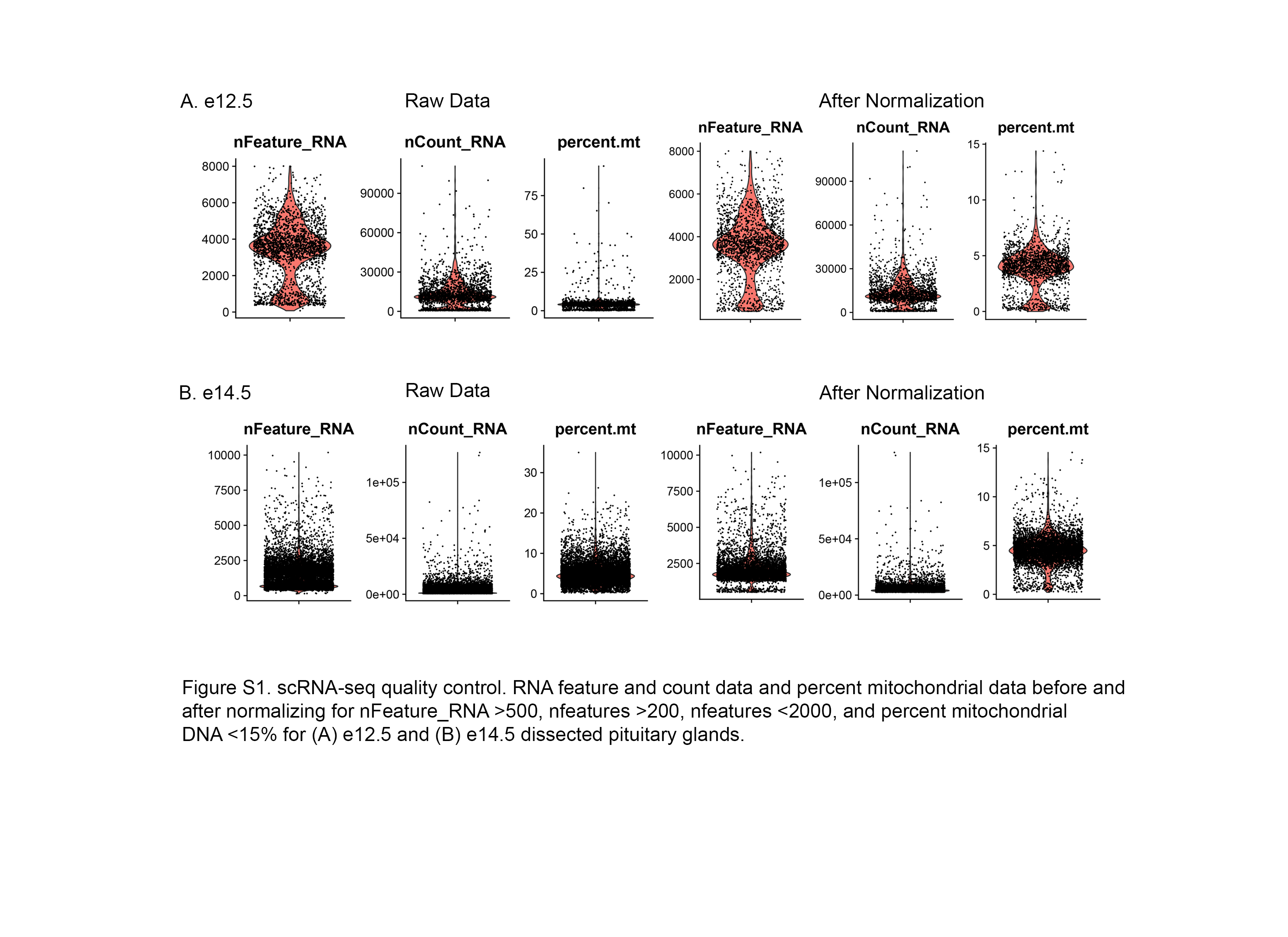

Supplement: Supplementary file 2 — Additional file 2: Figure S1. scRNA-seq quality control data. [file 13073_2024_1347_MOESM2_ESM.tif]

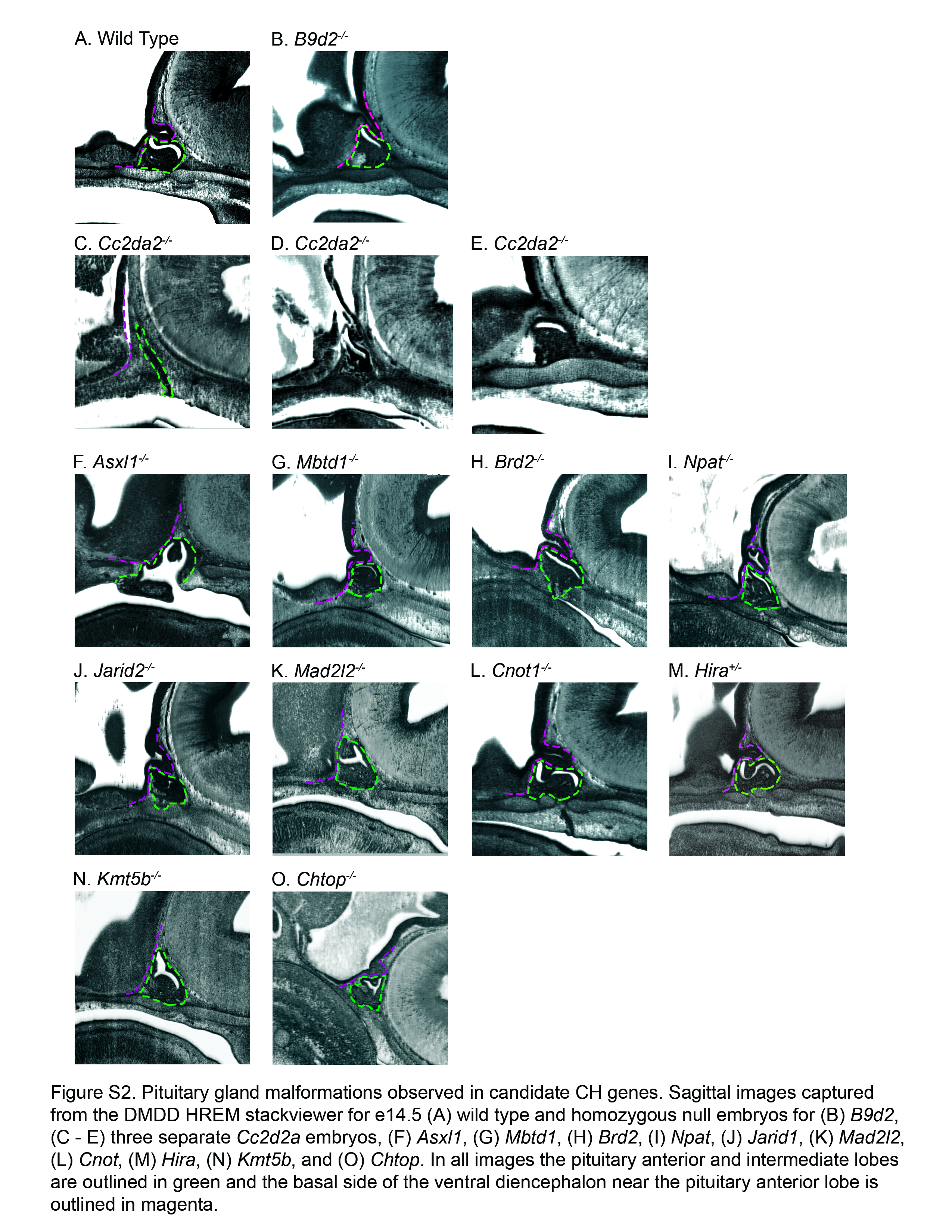

Supplement: Supplementary file 5 — Additional file 5: Figure S2. Pituitary gland malformations observed in candidate CH genes. [file 13073_2024_1347_MOESM5_ESM.tif]

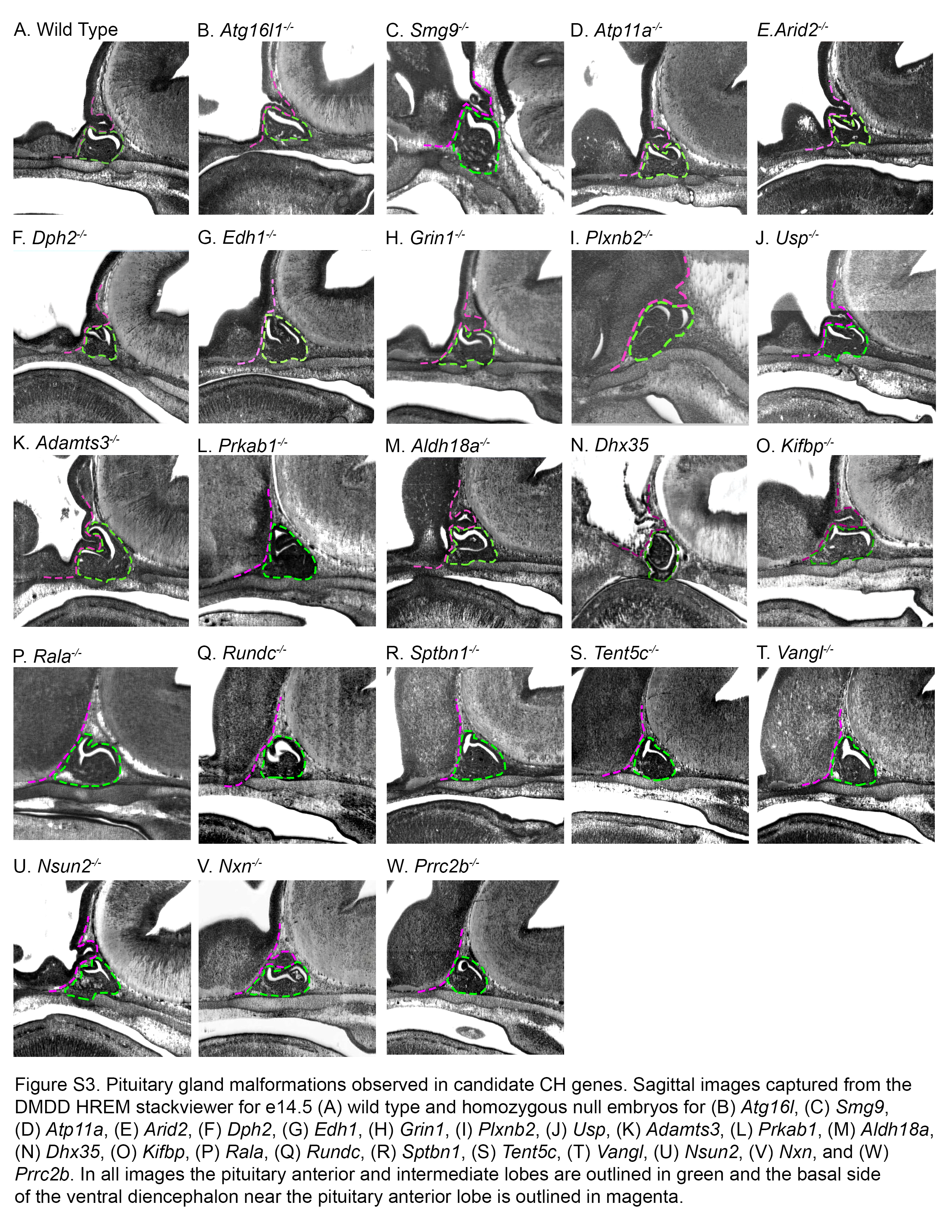

Supplement: Supplementary file 6 — Additional file 6: Figure S3. Pituitary gland malformations observed in candidate CH genes. [file 13073_2024_1347_MOESM6_ESM.tif]

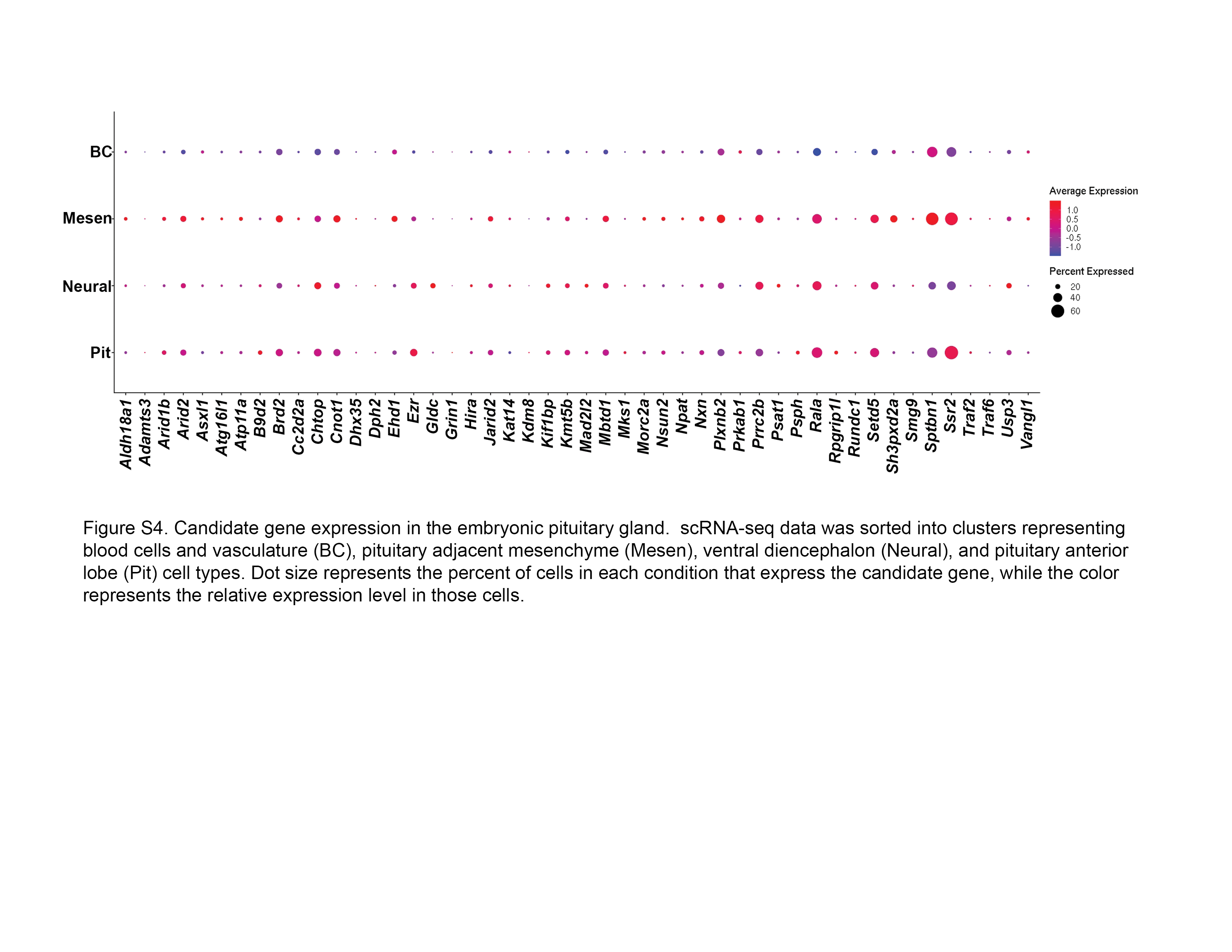

Supplement: Supplementary file 7 — Additional file 7: Figure S4. Candidate gene expression in the embryonic pituitary gland. [file 13073_2024_1347_MOESM7_ESM.tif]

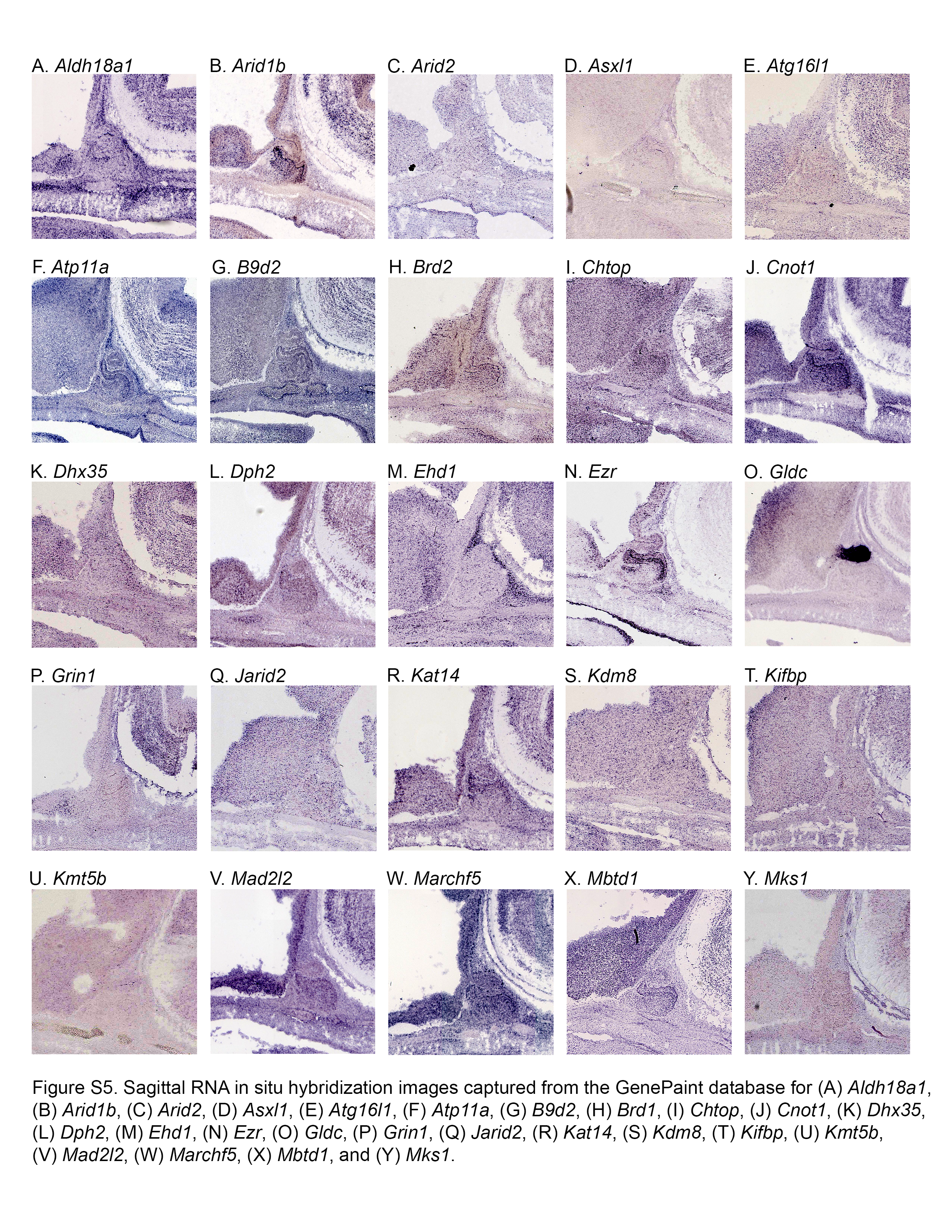

Supplement: Supplementary file 8 — Additional file 8: Figure S5. RNA in situ hybridization images captured from the GenePaint database. [file 13073_2024_1347_MOESM8_ESM.tif]

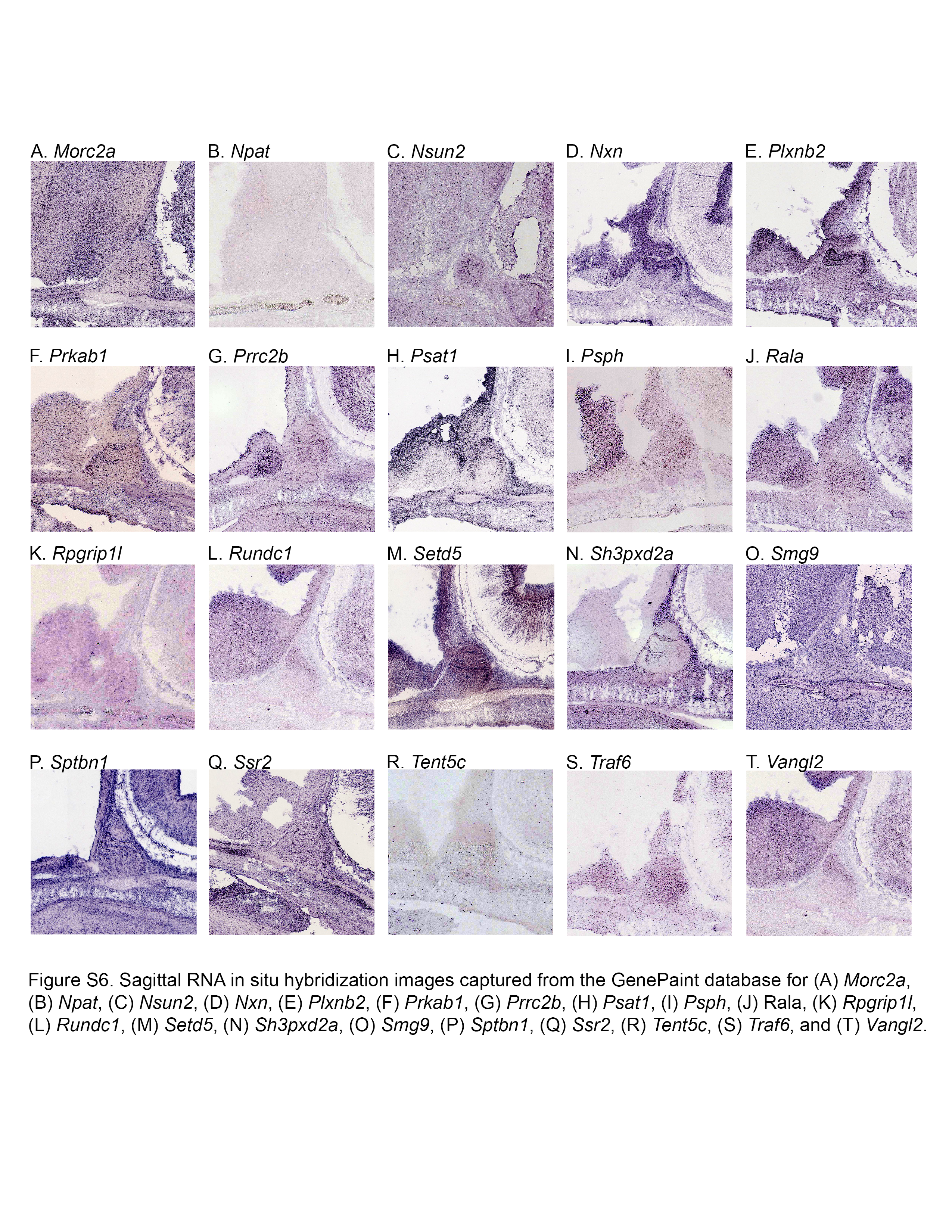

Supplement: Supplementary file 9 — Additional file 9: Figure S6. RNA in situ hybridization images captured from the GenePaint database. [file 13073_2024_1347_MOESM9_ESM.tif]
